# Reactions of Mesityl Azide with Ferrocene‐Based N‐Heterocyclic Germylenes, Stannylenes and Plumbylenes, Including PPh_2_‐Functionalised Congeners

**DOI:** 10.1002/chem.202200996

**Published:** 2022-06-08

**Authors:** Robin Guthardt, Lisa Oetzel, Tobias Lang, Clemens Bruhn, Ulrich Siemeling

**Affiliations:** ^1^ Institute of Chemistry University of Kassel Heinrich-Plett-Straße 40 34132 Kassel Germany; ^2^ Present address: School of Chemistry Monash University PO Box 23 VIC 3800 Melbourne Australia

**Keywords:** Azides, carbene homologues, metallocenes, subvalent compounds, tetrylenes

## Abstract

The reactivity of ferrocene‐based N‐heterocyclic tetrylenes [{Fe(η^5^−C_5_H_4_−NSi*t*BuMe_2_)_2_}E] (E=Ge, Sn, Pb) towards mesityl azide (MesN_3_) is compared with that of PPh_2_‐functionalised congeners exhibiting two possible reaction sites, namely the E^II^ and P^III^ atom. For E=Ge and Sn the reaction occurs at the E^II^ atom, leading to the formation of N_2_ and an E^IV^=NMes unit. The germanimines are sufficiently stable for isolation. The stannanimines furnish follow‐up products, either by [2+3] cycloaddition with MesN_3_ or, in the PPh_2_‐substituted case, by NMes transfer from the Sn^IV^ to the P^III^ atom. Whereas [{Fe(η^5^−C_5_H_4_−NSi*t*BuMe_2_)_2_}Pb] and other diaminoplumbylenes studied are inert even under forcing conditions, the PPh_2_‐substituted congener forms an addition product with MesN_3_, thus showing a behaviour similar to that of frustrated Lewis pairs. The germylenes of this study afford copper(I) complexes with CuCl, including the first structurally characterised linear dicoordinate halogenido complex [CuX(L)] with a heavier tetrylene ligand L.

## Graphical abstract

**The active Pb^II^/P Lewis pair nature** of a PPh_2_‐functionalised ferrocene‐based N‐heterocyclic plumbylene is demonstrated by its reaction with mesityl azide to afford a phosphazide, which is in contrast to the inertness of the unfunctionalised congener. The lighter analogues most likely react with MesN_3_ at their respective tetrel atom E^II^, leading to the formation of N_2_ and an E^IV^=NMes unit, followed by NMes transfer from the E^IV^ to the P^III^ atom.

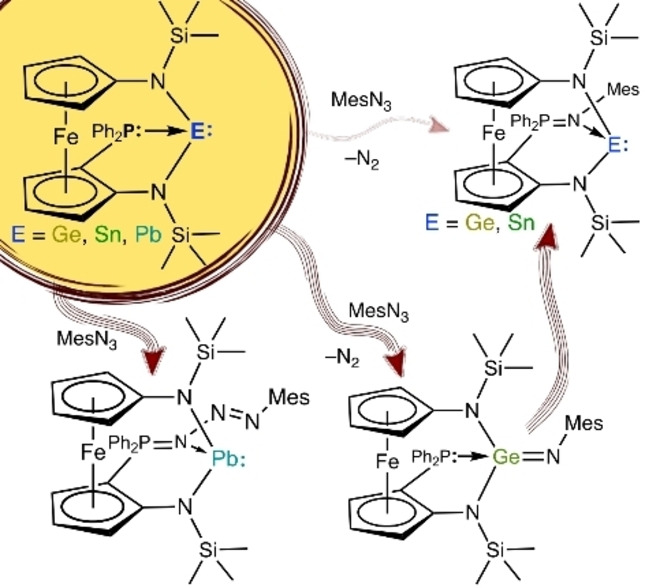

## Introduction

Since more than a century,^[1]^ the Staudinger reaction of organic azides RN_3_ with phosphanes R’_3_P (R, R’=alkyl or aryl) has provided access to iminophosphoranes RN=PR’_3_ via intermediate phosphazides RN=N−N=PR’_3_, which are generally unstable, but could be isolated in certain cases.^[2]^ A significant kinetic stabilisation of phosphazides by intramolecular coordination of the P‐bonded nitrogen atom to a Lewis acid was suggested by Grützmacher in 1999.^[3]^ In view of the well‐known analogy between phosphanes and N‐heterocyclic carbenes (NHCs),^[4]^ it is not surprising that organic azides can react with NHCs in a similar manner to afford cyclic guanidine derivatives NHC=NR, as was shown by Bielawski in 2005.^[5]^ The intermediate triazenes NHC=N−N=NR are thermally much more robust towards N_2_ extrusion than the analogous phosphazides RN=N−N=PR’_3_.^[6]^ We note in this context that the synthesis of imines of the type RN=CCl_2_ (i. e. isocyanide dichlorides)^[7]^ by reaction of organic azides with the transient singlet carbene Cl_2_C^[8]^ had been reported by Baldwin already in 1968; N_2_ extrusion occurs even below room temperature in this case.^[9]^ The heavier carbene analogues can react with organic azides in a similar fashion, giving rise to imine analogues with formal E=N (E=Si–Pb) double bonds.^[10]^ This was first shown by Satgé in 1978, who reported the formation of the transient germanimine (Me_2_N)_2_Ge=NPh together with N_2_ in the reaction of the diaminogermylene (Me_2_N)_2_Ge with PhN_3_.^[11]^ In 1991 Meller described the first structurally characterised stable germanimines [(Me_3_Si)ArN]_2_Ge=NAr [obtained from the reaction of ArN_3_ with [(Me_3_Si)ArN]_2_Ge, Ar=mesityl (Mes) or 2,6‐diisopropylphenyl (Dipp)].^[12]^ This was followed in 1993 by the first structurally characterised stable stannanimine [(Me_3_Si)_2_N]_2_Sn=NDipp (obtained from DippN_3_ and [(Me_3_Si)_2_N]_2_Sn at −30 °C).^[13]^ The only other stable stannanimine known to date, [(Me_3_Si)_2_CH]_2_Sn=N[Si*t*Bu_2_(N_3_)] [obtained from [(Me_3_Si)_2_CH]_2_Sn and Si*t*Bu_2_(N_3_)_2_], was reported one year later by Ando.^[14]^ The paucity of isolable stannanimines is due to the fact that, owing to their reactive polar Sn=N bond, stannanimines readily form their head‐to‐tail dimers (viz. 1,3,2,4‐diazadistannetidines) or give [2+3] cycloadducts with the organic azide to furnish stannatetrazoles, as was shown by Pinchuk and by Neumann already in the 1980s.^[15]^ The publication of the N‐heterocyclic silylene (CHN*t*Bu)_2_Si, the first stable compound containing dicoordinate Si^II^, by Denk and West in 1994^[16]^ was followed in the same year by their report of its reaction with trityl azide, which furnished the THF complex of the silanimine (CHN*t*Bu)_2_Si=NCPh_3_.^[17]^ Since then, numerous reactions of organic azides with free or base‐stabilised silylenes furnishing free or base‐stabilised silanimines have been described.[^10b^, ^10c^, ^18^] Among the reactions of organic azides with heavier carbene analogues, plumbylenes have apparently been investigated least extensively. We are aware of only three studies in this context.

**Scheme 1:**
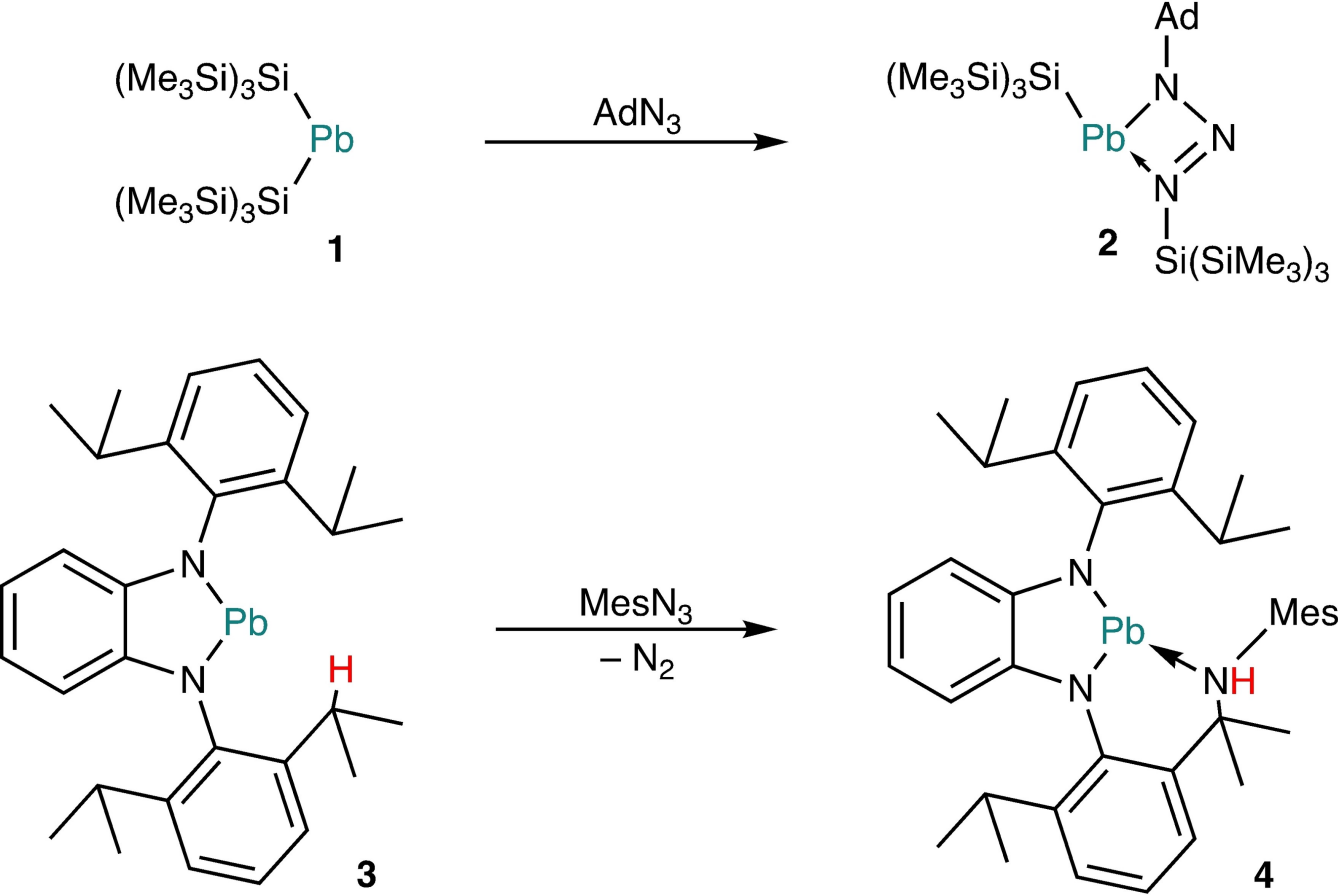
Reactions of organic azides with plumbylenes described by Klinkhammer (top) and Song (bottom); Ad=1‐adamantyl, Mes=mesityl.

The first study was published in 2002 by Klinkhammer, who described the reaction of [(Me_3_Si)_3_Si]_2_Pb (**1**) with 1‐adamantyl azide in toluene at −30 °C, which furnished the triazenido Pb^II^ complex **2** with a four‐membered PbN_3_ heterocycle due to migration of a (Me_3_Si)_3_Si group from Pb to N (Scheme 1, top).^[19]^ In 2017 Song reported the reaction of the N‐heterocyclic plumbylene *o*‐C_6_H_4_(NDipp)_2_Pb (**3**) with mesityl azide under harsh conditions (110 °C, toluene). N_2_ was liberated, but no plumbanimine was observed. The reaction resulted in a donor‐functionalised plumbylene **4** with tricoordinate Pb^II^ due to a chelating secondary amino group, which was plausibly formed by insertion of an NMes unit into a benzylic C−H bond, thus affording a Me_2_C−NH−Mes moiety (Scheme 1, bottom).^[20]^

**Scheme 2:**
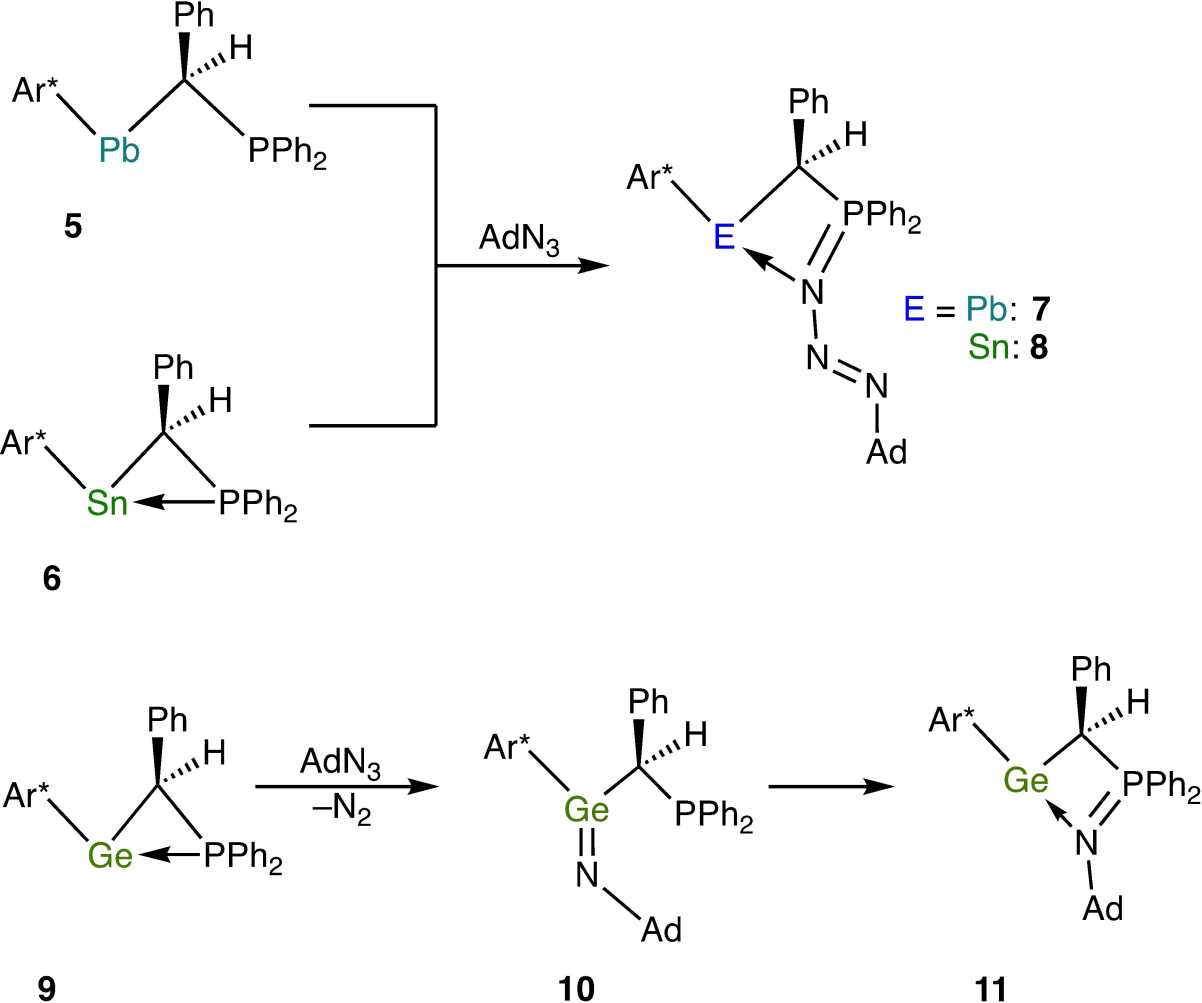
Reactions of 1‐adamantyl azide with PPh_2_‐functionalised tetrylenes described by Wesemann; Ar*=2,6‐(2,4,6‐*i*Pr_3_C_6_H_2_)_2_C_6_H_3_, Ad=1‐adamantyl.

**Figure 1:**
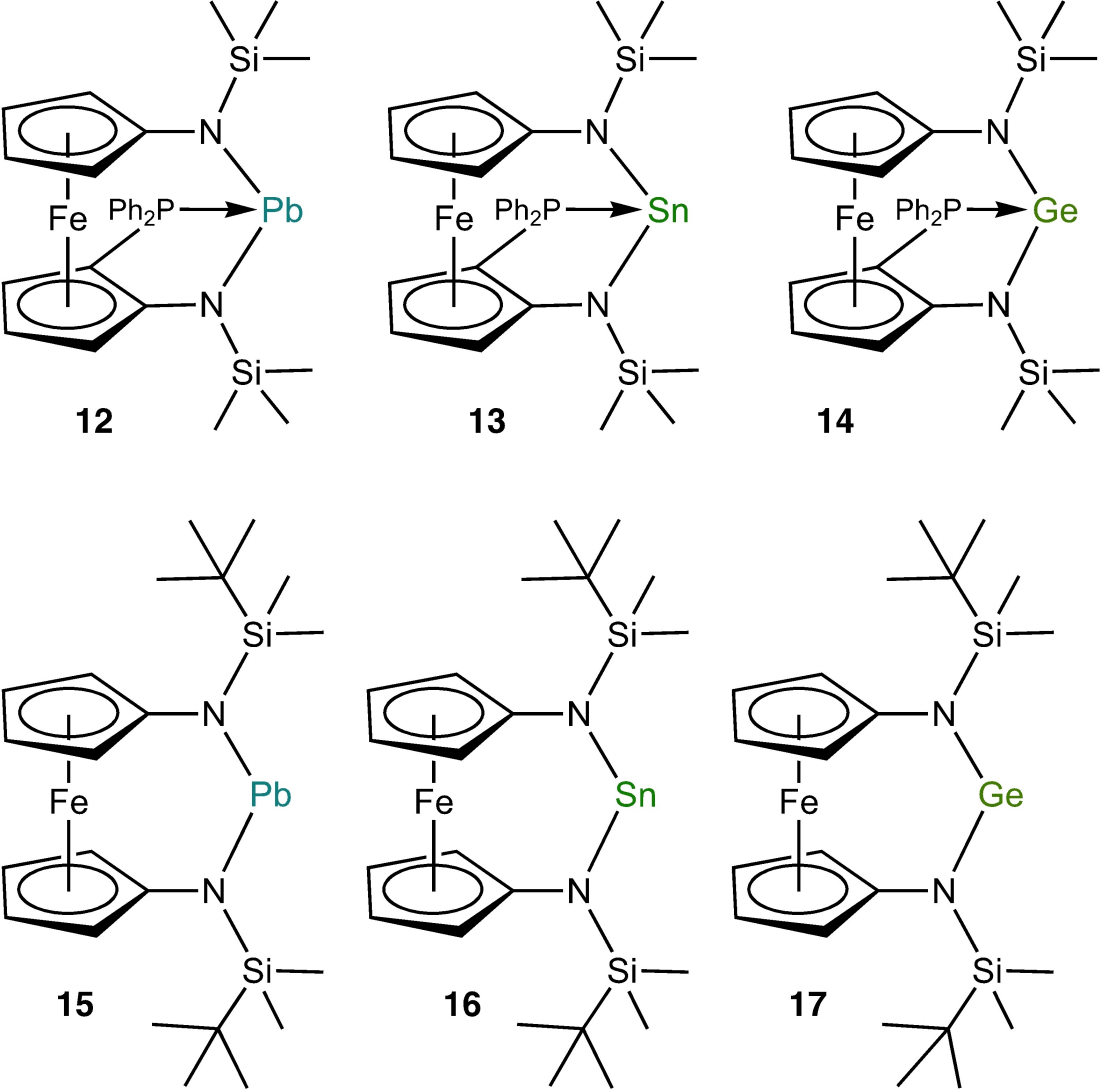
PPh_2_‐functionalised ferrocene‐based N‐heterocyclic tetrylenes (top) and unfunctionalised congeners (bottom) investigated in this study.

Of particular interest for the present work is Wesemann's study of the reaction of organic azides with intramolecular Lewis pairs composed of a tetrylene and a phosphane unit, which included also the plumbylene Ar*Pb[CHPh(PPh_2_)] [**5**, Ar*=2,6‐(2,4,6‐*i*Pr_3_C_6_H_2_)_2_C_6_H_3_] (Scheme 2).^[21]^ The Pb^II^ atom of **5** is dicoordinate. In contrast, the corresponding stannylene **6** ^[22]^ and germylene **9** ^[21]^ exhibit tricoordinate tetrel atoms due to intramolecular coordination of the P atom, thus resembling our recently reported ferrocene‐based N‐heterocyclic plumbylene **12**, stannylene **13** and germylene **14** functionalised with a PPh_2_ group (Figure 1, top).^[23]^

Compounds **5**, **6**, **9** and **12**–**14** contain two different sites suitable for reaction with an organic azide, viz. the P atom and the tetrel atom. Wesemann observed that plumbylene **5** and stannylene **6** react with AdN_3_ in the same fashion, affording addition products **7** and **8** featuring a four‐membered E*N*PC heterocycle (E=Sn, Pb; *N* denotes the terminal AdN_3_ nitrogen atom; Scheme 2, top). An analogous reaction had previously been reported by Ionkin for the stannylene [*t*Bu_2_PCH_2_C(CF_3_)_2_O]_2_Sn, which contains a tetracoordinate Sn^II^ atom due to intramolecular P coordination; only one equivalent of AdN_3_ was consumed even under forcing conditions.^[24]^ From a formal point of view, the products of these reactions can be described as phosphazides which are engaged in an intramolecular coordination of their P‐bonded nitrogen atom to the divalent tetrel atom. A completely different behaviour was found for germylene **9**, where formation of N_2_ and of the corresponding germanimine **10** (kinetic product] was observed, followed by slow isomerisation of the latter to the iminophosphorane‐functionalised germylene **11** (thermodynamic product; Scheme 2, bottom).^[21]^ The reactions of the Lewis pairs **5** and **6** with 1‐adamantyl azide are reminiscent of reactions reported for several borane‐phosphane (B/P) frustrated Lewis pairs (FLPs) with organic azides, where FLP addition to the terminal nitrogen atom of the azide (*N*) occurred, resulting in four‐ or five‐membered heterocycles with a B*N*P subunit.^[25]^ Wesemann's results inspired us to investigate the behaviour of plumbylene **12**, stannylene **13** and germylene **14** towards an organic azide. We selected MesN_3_, which is a readily available aryl azide considered particularly safe for use (C/N atom number ratio not lower than 3).^[26]^ We were interested in the influence of the Lewis pair nature of the donor‐functionalised N‐heterocyclic tetrylenes **12**–**14** in this context and therefore included the unfunctionalised congeners [{Fe(η^5^−C_5_H_4_−NSi*t*BuMe_2_)_2_}E] [E=Pb (**15**),^[27]^ Sn (**16**),^[28]^ Ge (**17**); Figure 1, bottom] in our study. The Si*t*BuMe_2_ substituent was chosen instead of the SiMe_3_ substituent present in **12**–**14**, because all three unfunctionalised tetrylenes are stable and can be isolated in pure form, whereas the trimethylsilyl‐substituted plumbylene [{Fe(η^5^−C_5_H_4_−NSiMe_3_)_2_}Pb] is unstable.^[27]^ Furthermore, the bulkier nature of Si*t*BuMe_2_ in comparison with SiMe_3_ is expected to compensate for part of the reduced steric encumbrance of the tetrel atom due to the absence of the PPh_2_ substituent.

## Results and Discussion

**Figure 2:**
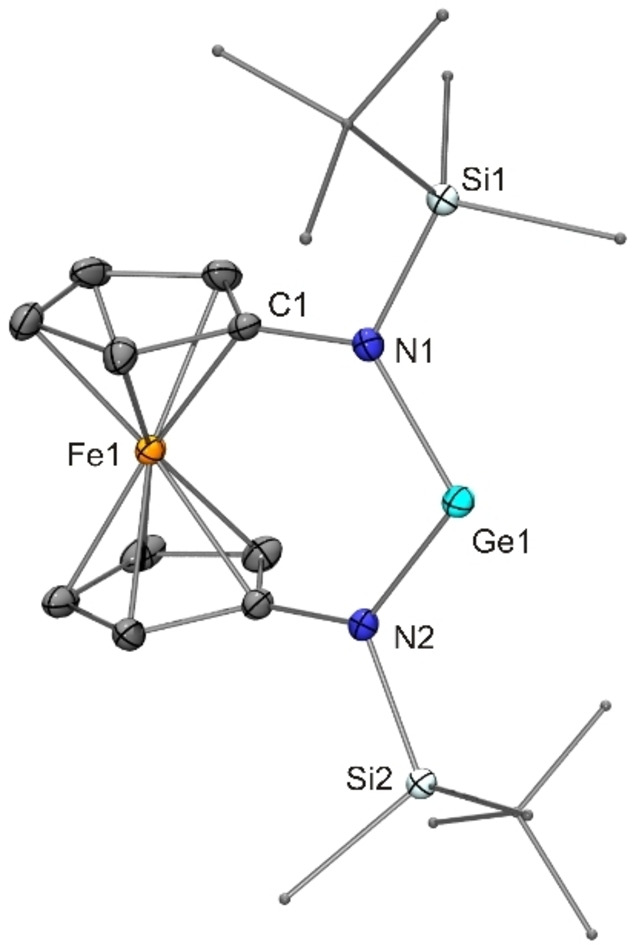
Molecular structure of germylene **17** in the crystal (ORTEP with ellipsoids drawn at the 50 % probability level, hydrogen atoms omitted for clarity, alkyl groups drawn as capped sticks).

The N‐heterocyclic germylene [{Fe(η^5^−C_5_H_4_−NSi*t*BuMe_2_)_2_}Ge] (**17**) was conveniently prepared analogous to the stannylene [{Fe(η^5^−C_5_H_4_−NSi*t*BuMe_2_)_2_}Sn] (**16**)^[28]^ by reacting LiN(SiMe_3_)_2_, [GeCl_2_(1,4‐dioxane)] and [Fe(η^5^−C_5_H_4_−NHSi*t*BuMe_2_)_2_] in a 2 : 1 : 1 molar ratio in THF. The product was obtained in 92 % yield and was structurally characterised by single‐crystal X‐ray diffraction (XRD). Pertinent metric parameters of germylene **17** and of the products of the reactions with MesN_3_ obtained in this study are collected in Table 1. Data for known N‐heterocyclic tetrylenes which served as starting materials for the new compounds contained in Table 1 have been included in this Table for comparison. The molecular structure of **17** is shown in Figure 2. The molecule exhibits approximate *C*_2_ symmetry about the Fe−Ge axis. The germanium bond lengths and angle are very similar to the values published for the *N*‐trimethylsilyl homologue.^[29]^ The germanium bond angle of 107.22(7)° determined for **17** is essentially identical to the value reported by Lappert for the emblematic acyclic diaminogermylene [(Me_3_Si)_2_N]_2_Ge, viz. 107.1(2)°.^[30]^ This is in line with previous observations that the 1,1’‐ferrocenylene backbone of N‐heterocyclic carbenes and their heavier analogues [{Fe(η^5^−C_5_H_4_−NR)_2_}E] (E=C–Pb) gives rise to large bond angles at the divalent tetrel atom close to those of corresponding acyclic congeners.[^27^, ^28^, ^29^, ^31^, ^32^] The Sn−N bonds of stannylene **16** are longer than the Ge−N bonds of germylene **17** by ca. 0.2 Å, which is in accord with the difference of the covalent radii of tin (1.39 Å) and germanium (1.20 Å).^[33]^ In turn, the Ge^II^ bond angle of **17** is slightly wider (by 2°) than the Sn^II^ bond angle of **16**, which is in agreement with Bent's rule.[^34^, ^35^]

**Table 1:** Pertinent metric parameters for the N‐heterocyclic tetrylenes of this study and the products of their reactions with mesityl azide.

	E−N1, E−N2 [Å]	E−N/P [Å]	N1−E−N2 [°]	Tilt angle [°]^[a]^	N−C_ipso_−C_ipso_−N [°]	
**12** (E=Pb)	2.260(2),	2.8624(8)	95.32(9)	6.3	26.2	ref. [23]
	2.213(2)					
**13** (E=Sn)	2.126(3),	2.7526(8)	98.11(10)	6.1	23.1	ref. [23]
	2.084(3)					
**14** (E=Ge)	1.9530(18),	2.6497(6)	100.69(7)	8.9	22.2	ref. [23]
	1.9160(18)					
**16** (E=Sn)	2.058(2),		105.09(10)	2.8	8.2	ref. [28]
	2.066(2)					
**17** (E=Ge)	1.8669(17),		107.22(7)	7.8	12.0	this work
	1.8685(18)					
**18** (E=Sn)^[b]^	2.035(5),	2.063(5)	115.60(19)	1.0	0.9	this work
	2.027(5)	2.072(5)				
**19** (E=Ge)^[c]^	1.830(5),	1.714(5)	114.6(2)	5.7	4.5	this work
	1.827(5)					
**20** (E=Pb)	2.308(3),	2.386(3)	91.19(11)	3.4	0.7	this work
	2.241(3)					
**21** (E=Sn)	2.174(3),	2.285(2)	94.05(10)	5.1	10.0	this work
	2.136(3)					
**22** (E=Ge)	1.8909(16),	1.7268(17)	108.06(7)	9.8	26.4	this work
	1.8625(16)	2.4535(5)				
**23** (E= Ge)	1.976(3),	2.110(3)	98.24(13)	7.7	11.1	this work
	1.943(3)					

[a] Dihedral angle formed by the best planes of the cyclopentadienyl rings. [b] Two independent molecules with very similar bond parameters; data arbitrarily given for molecule 1. [c] Two independent molecules, one of them showing disorder; data given for the non‐disordered molecule.

**Scheme 3:**
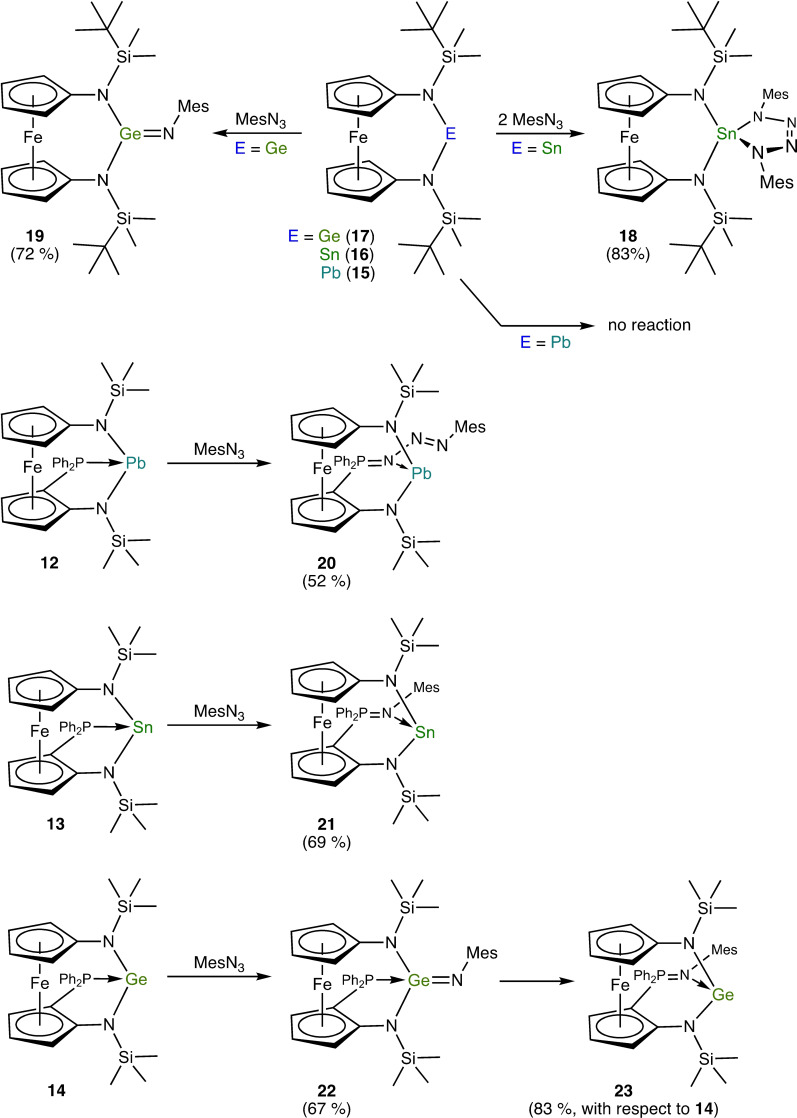
The ferrocene‐based N‐heterocyclic tetrylenes **15**–**17** and the Ph_2_P‐functionalised congeners **12**–**14** (used as racemic compounds, only one enantiomer is shown) and their reactions with mesityl azide (isolated yield given in brackets).

**Figure 3:**
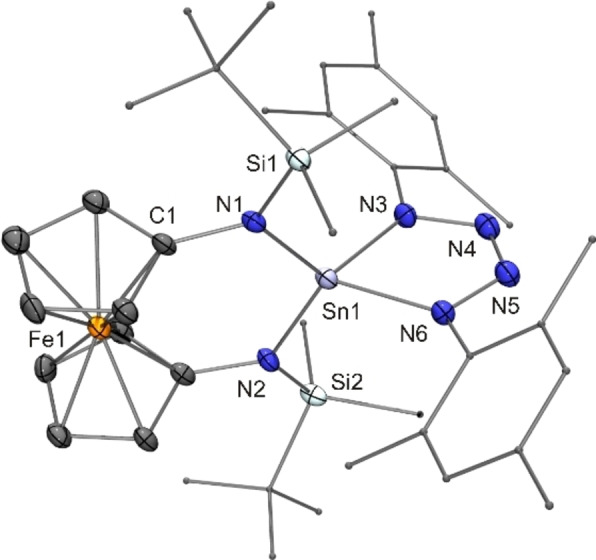
Molecular structure of stannatetrazole **18** in the crystal (ORTEP with ellipsoids drawn at the 50 % probability level, hydrogen atoms omitted for clarity, alkyl and aryl groups drawn as capped sticks). Selected bond lengths [Å] and angle [°]: N3−N4 1.390(7), N4−N5 1.267(8), N5−N6 1.396(7); N3−Sn1−N6 106.9(2).

With the complete series of donor‐functionalised heavier N‐heterocyclic tetrylenes **12**–**14** and the unfunctionalised congeners **15**–**17** in hand, we next studied the reactivity of these six compounds towards mesityl azide (Scheme 3). Reactions were observed for all compounds except [{Fe(η^5^−C_5_H_4_−NSi*t*BuMe_2_)_2_}Pb] (**15**). Since plumbylene **15** was inert even under rather forcing conditions (105 °C, toluene) almost identical to those used by Song for *o*‐C_6_H_4_(NDipp)_2_Pb (**3**; Scheme 1, bottom), we tested three additional diaminoplumbylenes in this context, viz. the acyclic congener [(Me_3_Si)_2_N]_2_Pb^[36]^ as well as our recently reported five‐ and six‐membered N‐heterocyclic plumbylenes *o*‐C_6_H_4_(NSiMe_3_)_2_Pb and nap(NSiMe_3_)_2_Pb (nap=naphthalene‐1,8‐diyl),^[31]^ all containing Me_3_Si instead of Si*t*BuMe_2_ substituents to decrease steric congestion. However, these compounds proved to be equally inert. The stannylene [{Fe(η^5^−C_5_H_4_−NSi*t*BuMe_2_)_2_}Sn] (**16**) showed a smooth and swift reaction with MesN_3_ (2 equiv.) at room temperature, furnishing the stannatetrazole **18** (structurally characterised by XRD, Figure 3) in 83 % yield. The use of 1 equiv. of the azide resulted in an equimolar mixture of **18** and unreacted **16**. This is in line with previous reports that an initially formed stannanimine is prone to form a [2+3] cycloadduct with an organic azide.[^13^, ^15d^, ^37^]

Stannatetrazole **18** is a spiro compound with a five‐ and a six‐membered heterocycle connected by the Sn atom. Schulz recently confirmed computationally that closely related compounds obtained from the acyclic diaminostannylene [(Me_3_Si)_2_N]_2_Sn and aryl azides contain a tin(IV) atom with four highly polar Sn−N single bonds instead of a tin(II) atom chelated by a tetraazabutadiene ligand.^[37a]^ A comparison of the Sn−N bond lengths of stannylene **16** (average value 2.06 Å, dicoordinate Sn^II^) and stannatetrazole **18** (average value 2.05 Å, tetracoordinate Sn^IV^) shows that obviously the increase in coordination number from two to four is compensated by the decrease in the covalent radius on going from Sn^II^ to Sn^IV^. This behaviour is not unusual. For example, a comparison of the cyclic diaminostannylene Me_2_Si(NDipp)_2_Sn^[38]^ and the corresponding tin(IV) spiro compound [Me_2_Si(NDipp)_2_]_2_Sn^[39]^ reveals essentially identical Sn−N bond lengths of 2.06 Å for both compounds. In agreement with the few structurally characterised stannatetrazoles known to date,[^37a^, ^37b^] the central N−N bond of the N_4_ unit of **18** is considerably shorter than the other two N−N bonds (1.27 vs. 1.39 Å), which is compatible with a double bond and adjacent single bonds, thus supporting the N−N=N−N Lewis structure advocated by Schulz.^[37a]^

**Figure 4:**
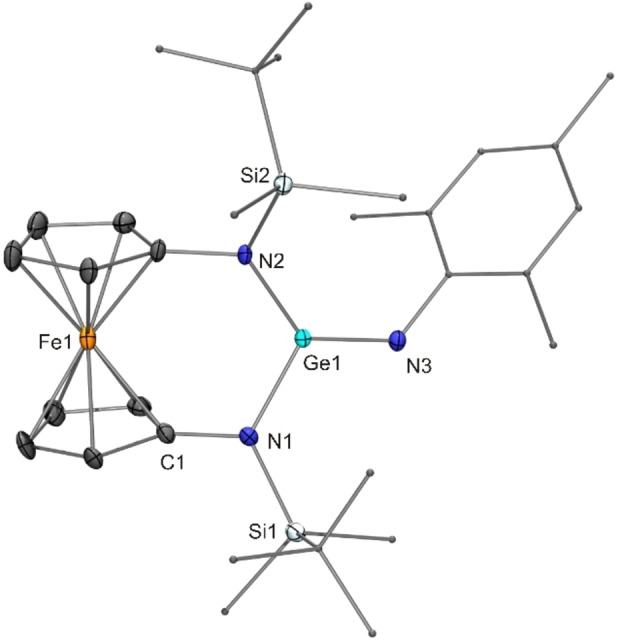
Molecular structure of the non‐disordered molecule of germanimine **19** in the crystal (ORTEP with ellipsoids drawn at the 50 % probability level, hydrogen atoms omitted for clarity, alkyl and aryl groups drawn as capped sticks).

In contrast to stannylene **16**, the corresponding germylene [{Fe(η^5^−C_5_H_4_−NSi*t*BuMe_2_)_2_}Ge] (**17**) afforded the germanimine [{Fe(η^5^−C_5_H_4_−NSi*t*BuMe_2_)_2_}Ge=NMes] (**19**; structurally characterised by XRD, Figure 4) in 72 % yield.[^12a^, ^40^] In comparison to stannanimines, the tendency of germanimines to undergo [2+3] cycloadditions with organic azides is less pronounced. For example, Meller found [2+3] cycloadduct (i. e. stannatetrazole) formation from the stannylene [(Me_3_Si)_2_N]_2_Sn and 2,6‐diethylphenyl azide at −50 °C,^[13]^ whereas Fulton observed germanimine formation from the corresponding germylene [(Me_3_Si)_2_N]_2_Ge and the significantly less bulky phenyl azide at −30 °C and with mesityl azide even at room temperature.^[40a]^ Germanimine **19** contains a trigonal‐planar Ge^IV^ atom (sum of angles 360°) exhibiting a short (1.71 Å) and two long Ge−N bonds (1.83 Å), in good agreement with germanimines derived from other diaminogermylenes.[^12a^, ^40^] In comparison to germylene **17**, the Ge−N single bonds of **19** are slightly shorter (by 0.04 Å). Similar to what was noted above for tin compounds **16** and **18**, the increase in coordination number from two to four is obviously outbalanced by the decrease in the covalent radius on going from Ge^II^ to Ge^IV^, in accord with findings of our recent systematic study addressing oxidation reactions of several homologues of **17**.^[41]^

**Figure 5:**
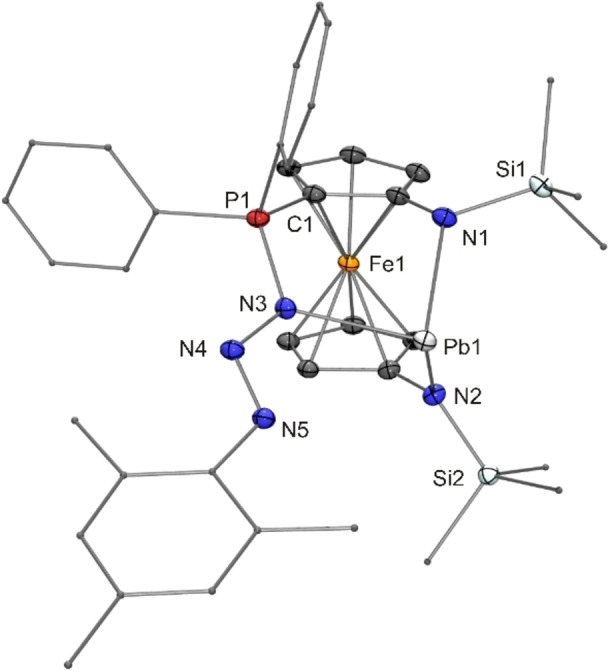
Molecular structure of phosphazide **20**⋅Et_2_O in the crystal (ORTEP with ellipsoids drawn at the 50 % probability level, hydrogen atoms and solvent molecule omitted for clarity, alkyl and aryl groups drawn as capped sticks). Selected bond lengths [Å]: P1−N3 1.643(3), N3−N4 1.375(4), N4−N5 1.274(4).

In contrast to [{Fe(η^5^−C_5_H_4_−NSi*t*BuMe_2_)_2_}Pb] (**15**), which was inert towards MesN_3_ even under forcing conditions (see above), the donor‐functionalised N‐heterocyclic plumbylene **12** reacted with this azide already under fairly mild conditions (60 °C, toluene), affording the addition product **20** (structurally characterised by XRD, Figure 5) with a six‐membered Pb*N*PCCN heterocycle (*N* denotes the terminal MesN_3_ nitrogen atom). **20** may be viewed as a phosphazide stabilised by intramolecular coordination of the P‐bonded nitrogen atom to the Lewis acidic Pb^II^ atom, which is in a trigonal pyramidal bonding environment (sum of angles 274°).

The Pb−NP bond is considerably longer (2.39 Å) than the two other Pb−N bonds (average value 2.27 Å). These values may be compared with the Pb−N distances of the 4‐dimethylaminopyridine (DMAP) adduct of plumbylene **15**,^[27]^ which exhibits two similarly short Pb−N bonds to the amino substituents (average value 2.27 Å), while the coordinative Pb−N_pyridine_ bond (2.50 Å) of [**15**(DMAP)] is ca. 11 Å longer than the Pb−NP bond of **20**, pointing to a significant ylidic character of the phosphazide unit (R’_3_P^+^−N^−^−N=NR) present in the latter. The Pb−NP bond length of Wesemann's plumbylene‐azide addition compound **7** has a value of 2.43 Å and is thus slightly longer (by 4 Å) than the corresponding bond of **20**, while their P−N bond lengths are essentially identical (1.64 Å).^[21]^

**Figure 6:**
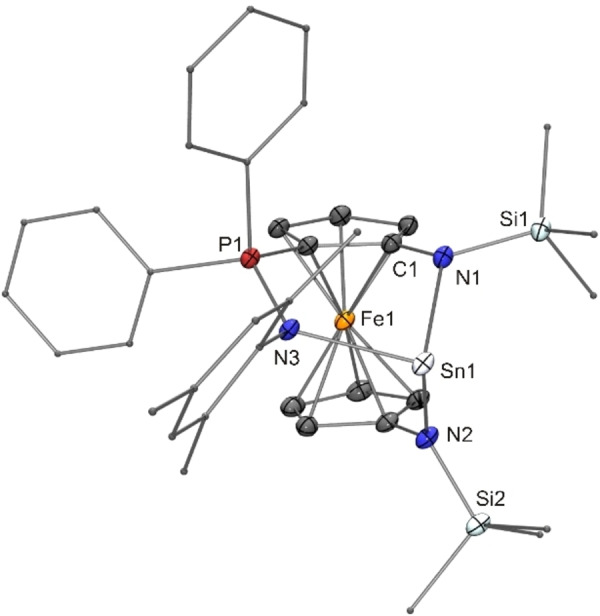
Molecular structure of the iminophosphorane‐functionalised stannylene **21** in the crystal (ORTEP with ellipsoids drawn at the 50 % probability level, hydrogen atoms omitted for clarity, alkyl and aryl groups drawn as capped sticks).

Whereas the reaction of [{Fe(η^5^−C_5_H_4_−NSi*t*BuMe_2_)_2_}Sn] (**16**) with MesN_3_ afforded a [2+3] cycloadduct (stannatetrazole **18**, see above), the donor‐functionalised congener **13** furnished the iminophosphorane‐functionalised stannylene **21** under the same mild conditions (structurally characterised by XRD, Figure 6).

The Sn^II^ atom of **21** is in a trigonal pyramidal bonding environment (sum of angles 289°). The Sn−NP bond is considerably longer (2.29 Å) than the two other Sn−N bonds (average value 2.16 Å) and essentially identical with the Sn−NP bond of the iminophosphorane‐functionalised diarylstannylene Ar*Sn{*o*‐C_6_H_4_[P(NAd)Ph_2_)]} reported by Wesemann; in the same vein, the P−N bond lengths of both compounds are indistinguishable within experimental error, viz. 1.619(3) Å for **21** and 1.613(5) Å for Wesemann's compound.^[42]^ A comparison of the tetrel‐nitrogen bonds present in **20** and **21** reveals that the bonds of stannylene **21** are shorter by ca. 0.1 Å than the corresponding bonds of plumbylene **20**, which is in line with the difference of the covalent radii of Sn (1.39 Å) and Pb (1.46 Å).^[33]^

In comparison to **12** and **13**, which contain tricoordinate P,N,N‐bonded tetrel atoms, the ^207^Pb NMR signal of **20** and the ^119^Sn NMR signal of **21** are moderately high‐field shifted in C_6_D_6_ solution [*δ*(^207^Pb)=2493 vs. 3050 ppm for **20** and **12**, respectively; *δ*(^119^Sn)=−1 vs. 187 ppm for **21** and **13**, respectively], in line with a tricoordinate N,N,N‐bonded nature of the respective tetrel atom.^[43]^ For comparison, a substantially low‐field shifted signal indicative of dicoordinate Pb^II^ and Sn^II^, respectively, was observed for [{Fe(η^5^−C_5_H_4_−NSiMe_3_)_2_}Pb] [*δ*(^207^Pb)=4333 ppm]^[27]^ and [{Fe(η^5^−C_5_H_4_−NSiMe_3_)_2_}Sn] [*δ*(^119^Sn)=589 ppm],^[29]^ which do not contain a donor substituent.

Finally, the reaction of mesityl azide with the donor‐functionalised germylene **14** at room temperature afforded the germanimine **22**, which was found to undergo a slow isomerisation to the corresponding iminophosphorane‐functionalised germylene **23**. Both isomers were structurally characterised by XRD.

**Figure 7:**
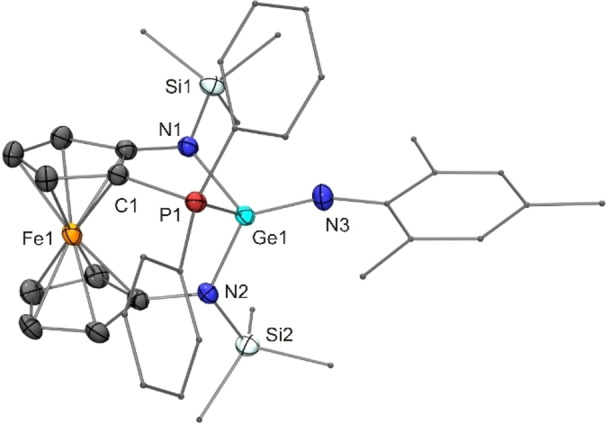
Molecular structure of germanimine **22** in the crystal (ORTEP with ellipsoids drawn at the 50 % probability level, hydrogen atoms omitted for clarity, alkyl and aryl groups drawn as capped sticks).

The PhP_2_‐functionalised germanimine **22** contains a tetracoordinate Ge^IV^ atom due to an intramolecular coordinative Ge−P bond (Figure 7), which is ca. 0.2 Å shorter than that of germylene **14**. Analogous to germanimine **19**, the Ge^IV^ atom is involved in a short (1.73 Å) and two long Ge−N bonds (average value 1.88 Å). These bonds are slightly elongated with respect to **19** (1.71 and 1.83 Å, see above) due to the higher coordination number of the Ge^IV^ atom of **22**, viz. four vs. three in **19**. Similar to **19**, Wesemann's germanimine **10**, which was obtained as the kinetic product from germylene **9** and AdN_3_, also contains a tricoordinate Ge^IV^ atom and exhibits a Ge−N bond length of 1.71 Å; however, no coordination of the PPh_2_ unit was observed in this case.^[21]^

**Figure 8:**
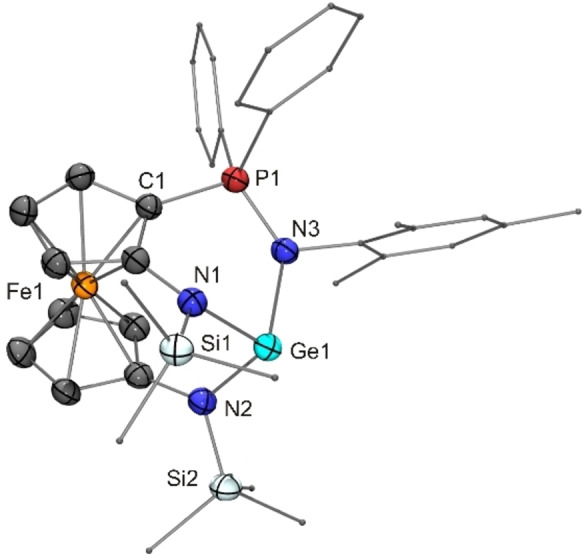
Molecular structure of the iminophosphorane‐functionalised germylene **23** in the crystal (ORTEP with ellipsoids drawn at the 50 % probability level, hydrogen atoms omitted for clarity, alkyl and aryl substituents drawn as capped sticks).

The iminophosphorane‐functionalised germylene **23** (Figure 8), which is formed as thermodynamic product from the PPh_2_‐functionalised germanimine **22** by rearrangement, contains a Ge^II^ atom in a trigonal pyramidal bonding environment (sum of angles 300°). The molecular structure is analogous to that of the corresponding stannylene **21**. The Ge−NP bond is considerably longer (2.11 Å) than the two other Ge−N bonds (average value 1.96 Å), in accord with the corresponding bond lengths determined for **21**, when the difference of the covalent radii of Sn (1.39 Å) and Ge (1.20 Å) is taken into account.^[33]^ The P−N bond lengths of both compounds are essentially identical, viz. 1.625(3) Å for **23** and 1.619(3) Å for **21**; they also compare well with the value of 1.608(2) Å reported by Wesemann for the iminophosphorane‐functionalised germylene **11**.^[21]^

When compared with the reactions of Wesemann's plumbylene **5** and germylene **9** with AdN_3_ (Scheme 2),^[21]^ the respective behaviour of plumbylene **12** and germylene **14** towards MesN_3_ is completely analogous. However, while Wesemann's PPh_2_‐functionalised stannylene **6** afforded an addition product (**8**), an iminophosphorane (**21**) was obtained from our PPh_2_‐functionalised stannylene **13**. The primary interaction of organic azides with main‐group element Lewis acids has been shown to involve the C‐bonded N atom.^[44]^ In contrast, the Staudinger reaction begins with a nucleophilic attack of the phosphane PR’_3_ on the terminal nitrogen atom (*N*) of the organic azide RN_3_.[^3b^, ^45^] Slootweg recently addressed the mechanism of the reaction of RN_3_ with the B/P FLP *t*Bu_2_PCH_2_BPh_2_ and found that the initial nucleophilic attack typical of a Staudinger reaction is kinetically less favourable than adduct formation of the Lewis acidic B atom with the Lewis basic C‐bonded N atom.^[25a]^ With MesN_3_ and *t*BuN_3_, the reaction afforded the respective addition product containing a four‐membered B*N*PC heterocycle via a six‐membered ring (BNN*N*PC) intermediate. The inertness of the unfunctionalised plumbylenes of our study towards MesN_3_ strongly indicates that the reaction of the azide occurs at the PPh_2_ unit of the donor‐functionalised plumbylenes **5** and **12**. The fact that **5** reacts already at room temperature, while elevated temperatures are needed in the case of **12** is perfectly plausible in view of the fact that the P atom of **12** is engaged in an intramolecular coordinative bond to the Pb atom, while no such bond is present in **5**, whose P atom is therefore readily available for reaction with the azide. In both cases, however, the resulting phosphazide is efficiently stabilised in this scenario by intramolecular adduct formation with the respective Lewis acidic Pb^II^ atom. Our results obtained with the lighter congeners, viz. stannylene pair **13** and **16** and germylene pair **14** and **17**, suggest that in these cases the reaction with the azide occurs at the divalent tetrel atom, leading to an E=N double bond, which is highly reactive in the case of E=Sn so that only follow‐up products (**18**, **21**) were observed.

**Scheme 4:**
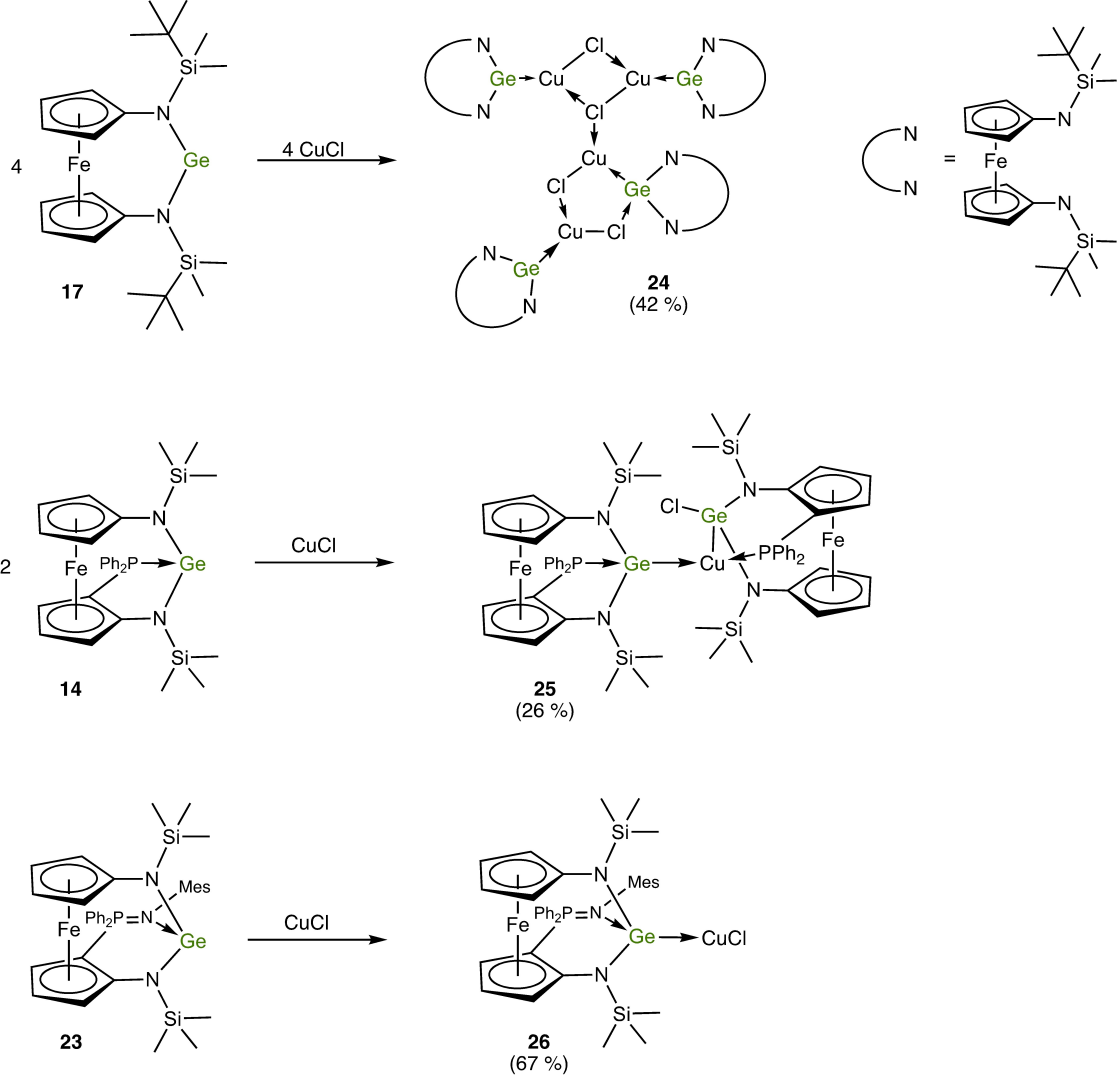
Reactions of the N‐heterocyclic germylenes **17**, **14** and **23** with CuCl (isolated yields given in brackets). The Lewis structures of the copper(I) complexes **24**, **25** and **26** reflect the results obtained for the solid state by XRD.

**Figure 9:**
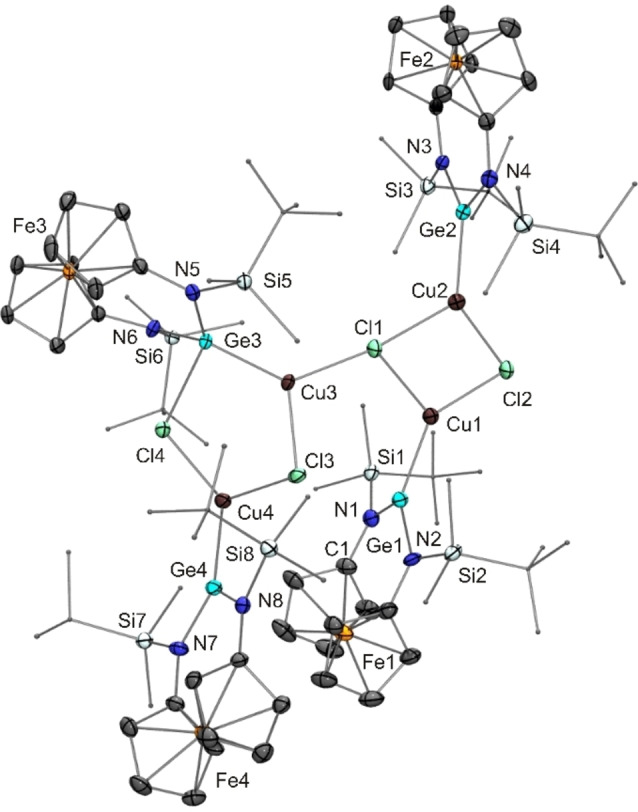
Molecular structure of copper(I) complex **24** in the crystal (ORTEP with ellipsoids drawn at the 50 % probability level, hydrogen atoms omitted for clarity, alkyl substituents drawn as capped sticks). Selected bond length [Å] and angle [°]: Ge3−Cl4 2.5991(14); Cl4−Ge3−Cu3 93.98(4).

Inspired in part by Breher's study of the ligand properties of the *N*‐mesityl homologue of **17** in transition metal chemistry^[46]^ as well as the recent report by Jambor and Herres‐Pawlis on copper(I) germylene complexes in the context of lactide polymerisation,^[47]^ we also addressed the coordination behaviour of the unfunctionalised germylene **17** and the donor‐functionalised congeners **14** and **23** towards CuCl (Scheme 4).^[48]^ Me_2_Si(N*t*Bu)_2_Ge appears to be the only cyclic diaminogermylene investigated in terms of CuCl coordination to date.^[48a]^ The complex obtained resulted from the reaction of six equivalents of this germylene with four equivalents of CuCl, thus exhibiting a 3 : 2 stoichiometric ratio of these components. In contrast to this, the reaction of **17** with CuCl furnished a product (**24**) with a composition corresponding to a 1 : 1 complex [(**17**)CuCl]. Although an NMR spectroscopic analysis of a C_6_D_6_ solution of this product revealed no significant coordination‐induced signal shifts in comparison to free **17**, an XRD study clearly showed that formation of a germylene‐copper(I) complex had taken place and confirmed the 1 : 1 ratio already inferred from microanalytical data. While the composition of product **24** corresponds to a simple 1 : 1 complex [(**17**)CuCl], the solid state structure is not that simple. The Lewis structure of **24** given in Scheme 4 corresponds to the molecular structure in the crystal, which is shown in Figure 9. Pertinent metric parameters of **24** and of the other copper(I) complexes of this study are collected in Table 2.

**Table 2:** Pertinent metric parameters for the copper complexes of this study.

	Ge−N^[a]^ [Å]	Ge−N/P [Å]	N−Ge−N^[a]^ [°]	Cu−Ge [Å]	Cu−Cl [Å]	
**24**	1.824(5),		111.2(2)	2.2561(9)	2.3459(16),^[b]^	
	1.832(5);				2.2417(15);	
	1.829(5),		112.0(2)	2.2504(9)	2.3838(15),^[b]^	
	1.834(5);				2.2253(17);	
	1.852(5),		107.4(2)	2.2881(8)	2.3078(14),^[b]^	
	1.861(4);				2.3000(16)	
	1.839(5),		110.7(2)	2.2983(9)	2.2972(14),	
	1.836(5)				2.2609(15)	
**25**	1.911(10),		102.0(4)	2.3817(19)		
	1.945(10);					
	1.946(8),	2.429(3)	100.8(4)	2.3530(19)		
	1.849(11)					
**26** ^[c]^	1.889(3),	1.979(3)	96.55(13)	2.2522(6)	2.1026(11)	
	1.878(3)				

[a] Cyclopentadienyl‐bonded N atoms. [b] Bond to tricoordinate Cl1. [c] Two independent molecules; data refer to the non‐disordered one.

The crystal structure of **24** exhibits four Cu^I^‐bonded germylene moieties in the asymmetric unit. Two of them form a chlorido‐bridged dimeric complex [(**17**)Cu(μ‐Cl)]_2_ containing two tricoordinate Ge^II^ atoms (Ge1 and Ge2) as part of a diamond‐shaped Cu_2_Cl_2_ core. The other two moieties form a less symmetric dimer, which contains one tricoordinate (Ge4) and one tetracoordinate Ge^II^ atom (Ge3). Only one of the two Cl atoms (Cl3) adopts a bridging position between the two Cu^I^ atoms of this dimer. The second Cl atom (Cl4) is in a bridging position between the tetracoordinate Ge^II^ atom Ge3 and the Cu^I^ atom Cu4 bonded to the tricoordinate Ge^II^ atom Ge4 of this less symmetric dimer. Instead of the diamond‐shaped Cu_2_Cl_2_ core of the other dimer, a five‐membered heterocyclic GeCu_2_Cl_2_ core is present in the less symmetric dimer. All four Cu^I^ atoms are in a trigonal planar coordination environment with two chlorine atoms and one germanium atom as bonding partners. The Cu^I^ atom Cu3 bonded to the tetracoordinate Ge^II^ atom Ge3 in the less symmetric dimer is connected to one of the Cl atoms (Cl1) of the Cu_2_Cl_2_ core of the symmetric dimer, thus joining the two dimeric units together and making this particular Cl atom μ_3_‐tricoordinate. The Cu−Cl distances of this tricoordinate Cl atom range from ca. 2.31 to 2.38 Å, while the other three Cl atoms exhibit shorter Cu−Cl bonds (2.23–2.30 Å) due to their dicoordinate nature. The Cu−Ge bond lengths of **24** lie in the small range from 2.25 to 2.30 Å, which compares well with other germylene complexes of tricoordinate Cu^I^.[^47^, ^48b^, ^48k^] The fact that Cl4 is bridging a Cu^I^ atom and a Ge^II^ atom, instead of two Cu^I^ atoms, suggests that the Cu−Cl and the Ge−Cl interactions in our system are of similar strength. Note that a chloride transfer to the germanium atom upon coordination of transition metal chlorides (MCl_2_, M=Fe, Co, Ni, Zn; CuCl), corresponding to the formation of a chlorogermyl ligand containing tetravalent germanium by Ge^II^ insertion into the M−Cl bond, was recently described by Cabeza for a donor‐stabilised N‐heterocyclic germylene with tricoordinate Ge^II^ due to intramolecular coordination of a P*i*Pr_2_ unit.[^48d^, ^48e^] The germanium atom of Cabeza's chlorogermyl copper complex is in a distorted pseudotetrahedral bonding environment, showing a distance of 0.75 Å to the plane formed by its two nitrogen atoms and the copper atom (sum of angles with respect to these three atoms: 321°).^[48e]^ The situation is quite different in the present case. Ge3 has a distance of only 0.23 Å from its CuN_2_ plane, close to a trigonal planar arrangement (sum of angles: 356°). The bond vector formed with its additional bonding partner, Cl4, is almost perpendicular to the CuN_2_ plane, in accord with a donor‐acceptor interaction of the chlorido ligand with the vacant p‐type orbital at the Ge^II^ atom. This notion is further supported by the Ge3−Cl4 distance of 2.60 Å. This bond is much longer than the Ge−Cl bonds of Cu^I^ complexes obtained from chlorogermylenes with tricoordinate Ge^II^ due to chelating β‐diketiminato or aminotropiminato units,[^48h^, ^48k^] which are typically 2.30 Å and thus close to the sum of the covalent radii of Ge (1.20 Å) and Cl (1.02 Å).^[33]^ In the same vein, the Cl−Ge−Cu angles in these chlorogermylene complexes are ca. 120°, while the Cl4−Ge3−Cu3 angle of **24** is 94°, reflecting the approximately perpendicular orientation of the Cl4−Ge3 bond vector with respect to the CuN_2_ plane as described above.

**Figure 10:**
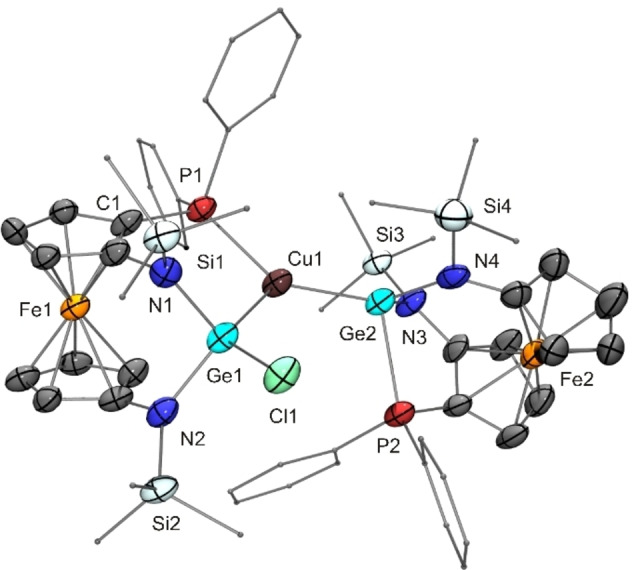
Molecular structure of copper(I) complex **25** in the crystal (ORTEP with ellipsoids drawn at the 50 % probability level, hydrogen atoms omitted for clarity, alkyl and aryl substituents drawn as capped sticks). Only one of the two enantiomers is shown. Selected bond lengths [Å]: Ge1−Cl1 2.261(3), Cu1−P1 2.269(3).

The reaction of **14** with CuCl afforded a product (**25**) with a composition corresponding to a 2 : 1 complex [(**14**)_2_CuCl] according to microanalytical data, which, however, were not in accord with an analytically pure sample of such composition. The product gave rise to rather complicated NMR spectra, which were not suitable to provide conclusive evidence for the nature of the species in solution. A structural investigation by XRD revealed a trigonal‐planar coordination environment of the Cu^I^ atom, which is bonded to a P atom and two different Ge atoms, one of them carrying the Cl atom. The Lewis structure of **25** given in Scheme 4 corresponds to the molecular structure in the crystal (Figure 10). The NMR spectra obtained for **25** are compatible with such a structure also in solution. Note that the tetravalent Ge atom Ge1 is a centre of chirality. Consequently, in combination with the two different planar‐chiral ferrocene moieties, four diastereomers may result. We have isolated only a single diastereomer, which was obtained as a racemic compound. Figure 10 arbitrarily shows the (*R*_p_,*R*,*S*_p_) enantiomer. The NMR spectra of the crude product do not indicate the presence of other diastereomers. In particular, the ^31^P{^1^H} NMR spectrum (Figure S26 in the Supporting Information) exhibits only two signals, as expected for **25** with its two different phosphorus atoms. **25** was obtained in only 26 % yield. We cannot exclude, therefore, that other diastereomers were also formed in the reaction of **14** with CuCl, but remained unnoticed due to low solubility.

The quality of the crystals obtained was poor (very small crystal size and weak scattering ability), thus compromising the result of the XRD analysis performed for **25**. Nevertheless, a meaningful discussion of metric parameters is possible at least for the heavy atoms. The Cu^I^ atom is in a trigonal planar bonding environment, being coordinated by two germanium atoms and one phosphorus atom. The Cu−P distance of 2.27 Å lies in the region typical for tricoordinate Cu^I^ triarylphosphane complexes, comparing well with, for example, [CuX_2_(PPh_3_)][NR_4_] (2.21 Å for X=Cl, R=Et; 2.24 Å for X=Br, R=*n*Bu; 2.23 Å for X=I, R=*n*Pr),^[49]^ [CuX(PPh_3_)_2_] (2.27, 2.28 and 2.27 Å for X=Cl, Br, I, respectively),^[50]^ [Cu(PPh_3_)_3_][BPh_4_] (2.26–2.29 Å),^[51]^ and [Cu{Ge(C_6_F_5_)_3_}(PPh_3_)_2_] (2.27 Å),^[52]^ the latter germyl complex apparently being the only structurally characterised tricoordinate Cu^I^ phosphane complex with a copper‐germanium bond (Cu−Ge 2.38 Å). Both germanium atoms of **25** are tetracoordinate and reside in a distorted pseudotetrahedral bonding environment. Their shared bonding partner is the Cu^I^ atom, the Cu−Ge bond length being 2.38 and 2.35 Å for Ge1 and Ge2, respectively. The intramolecular coordination of the P atom present in germylene **14** has remained intact for Ge2, but not for Ge1. Ge1 is tetravalent. The Cu−Ge1 (2.38 Å), Ge1−Cl (2.26 Å) and Ge1−N distances (average value 1.93 Å) are similar to those reported for Cabeza's chlorogermyl copper complex (see above; Cu−Ge 2.36 Å, Ge−Cl 2.26 Å, Ge−N 1.93 Å).^[39]^ The same holds true for the distance of the Ge atom to the CuN_2_ plane, which is 0.61 Å for Ge1 in **25** and 0.75 Å for Cabeza's compound (see above). However, the corresponding distance of Ge2 is only 0.29 Å. Ge2 is part of still intact germylene **14** acting as a ligand for the chlorogermyl‐bonded Cu^I^ atom. The loss of electron density at Ge2 by copper(I) complexation obviously leads to a stronger coordination of the PPh_2_ moiety, as is reflected by a Ge2−P2 distance of 2.43 Å as opposed to 2.65 Å determined for the Ge−P bond of germylene **F**; a much smaller, but still significant, contraction is observed for the corresponding Ge−N bonds (average value 1.93 Å in **14** vs. 1.90 Å for Ge2 in **25**). A similar effect, albeit less pronounced, has been observed by Baceiredo for a donor‐stabilised germylene with tricoordinate Ge^II^ due to intramolecular coordination of a PPh_2_ unit, whose Ge−P distance shortens from 2.43 to 2.39 Å upon complexation by a {RhCl(COD)} fragment.^[53]^ To summarise, compound **25** contains a tetravalent germanium atom (Ge1, chlorogermyl ligand) and a divalent germanium atom (Ge2, donor‐stabilised germylene ligand). To a first approximation, bonding in this complex may be rationalised, and symbolised, in the following simplified way: P:→Ge:→Cu−Ge.^[54]^

**Figure 11:**
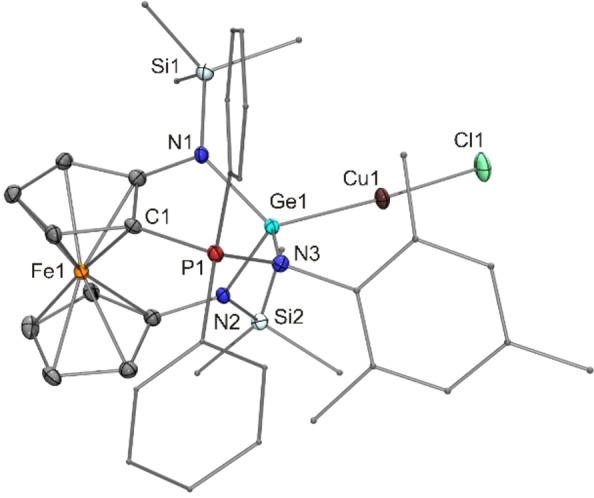
Molecular structure of copper(I) complex **26** in the crystal (ORTEP with ellipsoids drawn at the 50 % probability level, hydrogen atoms omitted for clarity, alkyl and aryl substituents drawn as capped sticks). Selected bond angle [°]: Ge1−Cu1−Cl1 176.24(4).

Finally, the reaction of the iminophosphorane‐functionalised germylene **23** with CuCl cleanly afforded the germylene‐copper(I) complex [(**23**)CuCl] (**26**, Scheme 4). In view of the substantial number of Cu^I^ iminophosphorane complexes,^[55]^ it has not been obvious a priori that this reaction leads to a Cu^I^ germylene complex. Solution NMR spectra are in accord with the structure found in the solid state, which is shown in Figure 11. The coordination environment of the Cu^I^ atom of **26** is linear dicoordinate (bond angle 176°). The Cu−Cl bond length of 2.10 Å is typical for this arrangement and several structurally characterised examples of complexes [CuCl(L)] with L=carbene (CAAC, NHC) or donor‐stabilised silylene, but not with L=germylene or heavier analogues, have been reported.^[56]^ The Cu−Ge distance of 2.25 Å corresponds to the shortest Cu−Ge bond lengths determined for **24** (see above), where we have a combination of tricoordinate Ge^II^ and tricoordinate Cu^I^. In the case of **26**, we have a combination of dicoordinate Cu^I^ and tetracoordinate Ge^II^, since the intramolecular coordination of the iminophosphorane N atom found for germylene **23** is also present in its copper complex **26**. Cu^I^ coordination leads to a substantial shortening of all three Ge−N bonds. Analogous to **25** (see above), the effect is largest (0.13 Å) for the coordinative bond, which is contracted from 2.11 Å in **23** to 1.98 Å in **26**, while the two other Ge−N bonds experience a less pronounced contraction (0.08 Å on average).

## Conclusion

We have compared the reactivity of the ferrocene‐based N‐heterocyclic tetrylenes [{Fe(η^5^−C_5_H_4_−NSi*t*BuMe_2_)_2_}E] [E=Pb (**15**), Sn (**16**),Ge (**17**)] towards mesityl azide with that of the PPh_2_‐functionalised congeners **12**–**14**, whose phosphorus(III) atom constitutes a second possible reaction site in addition to the respective tetrel(II) atom. Our results indicate that the reaction of this azide with the stannylenes **13** and **16** and germylenes **14** and **17** invariably occurs at the divalent tetrel atom, leading to an E=N double bond. The resulting germanimines **19** and **22** could be isolated. However, the latter isomerised readily to iminophosphorane **23** by NMes transfer from the Ge^IV^ to the P^III^ atom due the rather reactive Ge=N bond. In line with previous observations (see above), the reactivity of the Sn=N bond is even higher, so that only follow‐up products were observed in the reactions of the stannylenes **13** and **16**, namely, iminophosphorane **21** (most likely formed by NMes transfer from the Sn^IV^ to the P^III^ atom of the transient stannanimine) and stannatetrazol **18** (formed by [2+3] cycloaddition of the transient stannanimine with mesityl azide). All four unfunctionalised diaminoplumbylenes of our study, viz. the N‐heterocyclic compounds *o*‐C_6_H_4_(NSiMe_3_)_2_Pb, nap(NSiMe_3_)_2_Pb and [{Fe(η^5^−C_5_H_4_−NSi*t*BuMe_2_)_2_}Pb] (**15**) as well as the acyclic congener [(Me_3_Si)_2_N]_2_Pb, proved to be inert towards MesN_3_ even under forcing conditions. In contrast, the behaviour of the PPh_2_‐functionalised diaminoplumbylene **12** towards mesityl azide is analogous to that of Wesemann's PPh_2_‐functionalised (alkyl)(aryl)plumbylene **5**, being strongly reminiscent of B/P FLPs in both cases. While **5** reacts already at room temperature, moderately higher temperatures are needed in the case of **12**. This may be ascribed to the fact that the P atom of **12**, in contrast to that of **5**, is engaged in an intramolecular coordinative bond to the Pb atom. Consequently, plumbylene **12** exhibits a reduced frustration in comparison to **5** and thus belongs to the so‐called active Lewis pairs (ALPs), which can show FLP‐like behaviour due to the weakness of their coordinative bond.^[57]^ Investigations addressing the activation of fundamentally important small molecules with **12** and closely related ALPs are underway in our laboratory. In addition, our study has demonstrated the ability of the unfunctionalised N‐heterocyclic germylene **17** and its donor‐functionalised relatives **14** and **23** to act as ligands for copper(I), thus underlining the potential of such ferrocene‐based, and hence redox‐functionalised, N‐heterocyclic germylenes in coordination chemistry.^[58]^ Notably, compound **26** is the first structurally characterised linear dicoordinate copper(I) halogenido complex [CuX(L)] with a heavier tetrylene ligand L.

## Experimental Section

All reactions involving air‐sensitive compounds were performed in an inert atmosphere (argon or dinitrogen) by using standard Schlenk techniques or a conventional glovebox. Starting materials were procured from standard commercial sources and used as received. [Fe(η^5^−C_5_H_4_−NHSi*t*BuMe_2_)_2_],^[59]^ [GeCl_2_(1,4‐dioxane)],^[60]^ **12**–**14**,^[23]^ **15**,^[27]^ **16** ^[28]^ and MesN_3_ ^[61]^ were synthesised by adapted versions of the published procedures. NMR spectra were recorded at ambient temperature with Varian NMRS‐500 and MR‐400 spectrometers operating at 500 and 400 MHz, respectively, for ^1^H. Elemental analyses were carried out with a HEKAtech Euro EA‐CHNS elemental analyser at the Institute of Chemistry, University of Kassel, Germany.

**Synthesis of 17**: LiN(SiMe_3_)_2_ (395 mg, 2.36 mmol) was added to a stirred solution of [Fe(η^5^−C_5_H_4_−NHSi*t*BuMe_2_)_2_] (500 mg, 1.12 mmol) in THF (8 mL). After 30 minutes [GeCl_2_(1,4‐dioxane)] (260 mg, 1.12 mmol) was added and stirring was continued for 2 h. Volatile components were removed under reduced pressure. *n*‐Hexane (6 mL) was added to the residue. Insoluble material was removed by filtration through a short pad of celite. The solvent was removed from the filtrate under reduced pressure, leaving the product as a brownish‐yellow viscous oil. Yield 534 mg (92 %). Crystallisation of the product was initialised in a concentrated *n*‐hexane solution by scratching with a glass rod. After the first crystals appeared, the flask was stored at −40 °C. The mother liquor was separated from the orange product, which was finally dried under reduced pressure. ^1^H NMR (400 MHz, C_6_D_6_): *δ*=3.87, 3.78 (2 m, 2×4 H, cyclopentadienyl H), 1.00 (s, 18 H, CMe_3_), 0.29 (s, 12 H, SiMe_3_) ppm. ^13^C{^1^H} NMR (101 MHz, C_6_D_6_): *δ*=110.1 (C_ipso_), 68.9, 66.7 (2×CH), 27.6 (C*Me*_3_), 18.9 (*C*Me_3_), −1.0 ppm (SiMe_2_). Anal. calcd for C_22_H_38_N_2_FeGeSi_2_ (515.20): C 51.29, H 7.43, N 5.44 %; found C 50.74, H 7.56, N 5.19 %.

**Synthesis of 18**: Mesityl azide (77 mg, 0.24 mmol) was added to a stirred solution of **16** (132 mg, 0.24 mmol) in *n*‐hexane (3 mL). Stirring was discontinued after 30 min. The solution was stored at −40 °C for crystallisation. The mother liquor was separated from the yellow crystals, which were subsequently dried under reduced pressure. Yield 168 mg (83 %). ^1^H NMR (400 MHz, C_6_D_6_): *δ*=6.93 (s, 4 H, Mes CH), 3.82, 3.73 (2 m, 2×4 H, cyclopentadienyl H), 2.83 (s, 12 H, Mes *o*‐CH_3_), 2.23 (s, 6 H, Mes *p*‐CH_3_), 0.62 (s, 18 H, CMe_3_), 0.09 ppm (s, 12 H, SiMe_2_). ^13^C{^1^H} NMR (101 MHz, C_6_D_6_): *δ*=142.7 (Mes C_ipso_), 131.9 (Mes CH), 128.8, 127.4 (2×Mes *C*CH_3_), 100.2 (cyclopentadienyl C_ipso_), 70.3, 67.7 (2×cyclopentadienyl CH), 27.2 (C*Me*_3_), 24.5 (Mes *o*‐CH_3_), 20.6 (Mes *p*‐CH_3_), 20.5 (*C*Me_3_), −2.6 ppm (SiMe_2_). ^119^Sn{^1^H} NMR (186 MHz, C_6_D_6_): *δ*=−217.7 ppm. Anal. calcd for C_40_H_60_N_6_FeSi_2_Sn (855.67): C 56.15, H 7.07, N 9.82 %; found C 56.34, H 7.01, N 8.88 %.

**Synthesis of 19**: A solution of mesityl azide (52 mg, 0.32 mmol) in *n*‐hexane (1 mL) was added to a stirred solution of **17** (165 mg, 0.32 mmol) in *n*‐hexane (5 mL). After 10 minutes the volume of the solution was reduced to ca. 3 mL. The solution was stored at −40 °C for crystallisation. The orange product was separated from the mother liquor and subsequently dried under reduced pressure. Yield 149 mg (72 %). ^1^H NMR (400 MHz, C_6_D_6_): *δ*=7.01 (s, 2 H, Mes CH), 3.91, 3.82 (2 m, 2×4 H, cyclopentadienyl H), 2.55 (s, 6 H, Mes *o*‐CH_3_), 2.30 (s, 3 H, Mes *p*‐CH_3_), 0.94 (s, 18 H, CMe_3_), 0.26 ppm (s, 12 H, SiMe_2_). ^13^C{^1^H} NMR (101 MHz, C_6_D_6_): *δ*=149.7 (Mes C_ipso_), 129.1 (Mes CH), 94.7 (cyclopentadienyl C_ipso_), 69.3, 68.5 (2×cyclopentadienyl CH), 27.5 (C*Me*_3_), 21.4 (Mes *o*‐CH_3_), 21.1 (*C*Me_3_), 20.1 (Mes *p*‐CH_3_), −2.8 ppm (SiMe_2_); Mes *C*CH_3_ not detected. Anal. calcd for C_31_H_49_N_3_FeGeSi_2_ (648.39): C 57.42, H 7.62, N 6.48 %; found C 58.40, H 6.86, N 6.44 %.

**Synthesis of 20**: A solution of mesityl azide (22 mg, 0.13 mmol) in toluene (2 mL) was added to a solution of **12** (100 mg, 0.13 mmol) in toluene (3 mL). The stirred mixture was heated to 60 °C for 10 h. Subsequently, volatile components were removed under reduced pressure. The crude product was dissolved in diethyl ether/*n*‐hexane (1 : 1, 2 mL) and the solution was stored at −40 °C for crystallisation. This afforded the product as red diethyl ether solvate, which was separated from the mother liquor and subsequently dried under reduced pressure. Yield 68 mg (52 %). ^1^H NMR (400 MHz, C_6_D_6_): *δ*=7.72–7.68, 7.67–7.60 (2 m, 2×2 H, Ph *o*‐H), 7.05–6.98 (m, 2 H, Ph *p*‐H), 6.98–6.87 (m, 4 H, Ph *m*‐H), 6.72 (s, 2 H, Mes CH), 4.29, 3.97 (2 m, 2×2 H, cyclopentadienyl H), 3.93, 3.65, 3.59 (3 m, 3×1 H, cyclopentadienyl CH), 2.25 (s, 6 H, Mes *o*‐CH_3_), 2.09 (s, 3 H, Mes *p*‐CH_3_), 0.48, 0.25 ppm (2 s, 2×9 H, SiMe_3_). ^13^C{^1^H} NMR (101 MHz, C_6_D_6_): *δ*=145.9 (Mes C_ipso_), 135.5 (Mes *p*‐*C*CH_3_), 133.6, 133.0 (2 d, *J*=10.2 Hz, Ph *o*‐CH), 132.9, 132.4 (2 d, *J*=2.9 Hz, Ph *p*‐CH), 131.0, 130.0 (Mes CH), 129.1 (second line of doublet concealed by solvent signal, Ph C_ipso_), 128.7 (Mes *o*‐*C*CH_3_), 128.6 (two very closely spaced signals, 2×Ph CH), 126.4 (d, ^1^ *J*=89.9 Hz, Ph C_ipso_), 122.3 (d, *J*=7.7 Hz), 115.9 (2×cyclopentadienyl C_ipso_N), 72.7 (d, *J*=10.9 Hz), 72.1, 69.4, 67.9 (d, *J*=16.7 Hz), 66.5 (d, *J*=14.3 Hz), 65.6, 63.0 (7×cyclopentadienyl CH), 55.4 (d, ^1^ *J*=130.3 Hz, cyclopentadienyl C_ipso_P), 20.9 (Mes *p*‐CH_3_), 20.2 (Mes *o*‐CH_3_), 3.3, 3.3 ppm (2×SiMe_3_). ^31^P{^1^H} NMR (202 MHz, C_6_D_6_): *δ*=38.4 ppm (^2^ *J*_PbP_=96 Hz). ^207^Pb NMR (105 MHz, C_6_D_6_): *δ*=2493 ppm. Anal. calcd for C_37_H_46_N_5_FePPbSi_2_⋅C_4_H_10_O (985.12): C 49.99, H 5.73, N 7.11 %; found C 50.64, H 5.43, N 7.41 %.

**Synthesis of 21**: A solution of mesityl azide (27 mg, 0.21 mmol) in *n*‐hexane (1 mL) was added to a stirred solution of **13** (110 mg, 0.17 mmol) in *n*‐hexane (3 mL). Stirring was discontinued after 10 min. The solution was stored at −40 °C for crystallisation. The mother liquor was separated from the orange crystals, which were subsequently dried under reduced pressure. Yield 91 mg (69 %). ^1^H NMR (500 MHz, C_6_D_6_): *δ*=7.97, 7.40 (2 m, 2×2 H, Ph *o*‐H), 7.06 (m, 1 H, Ph *p*‐H), 6.97 (m, 2 H, Ph *m*‐H), 6.92 (m, 1 H, Ph *p*‐H), 6.84 (m, 2 H, Ph *m*‐H), 6.73, 6.63 (2 s, 2×1 H, Mes CH), 4.61, 4.42, 4.05, 3.95, 3.90, 3.70, 3.47 (7 m, 7×1 H, cyclopentadienyl H), 2.43 (d, *J*=1.8 Hz, 3 H, Mes CH_3_), 2.09 (d, *J*=2.4 Hz, 3 H, Mes CH_3_), 1.83 (d, *J*=1.9 Hz, 3 H, Mes CH_3_), 0.53, 0.43 ppm (2 s, 2×9 H, SiMe_3_). ^13^C{^1^H} NMR (101 MHz, C_6_D_6_): *δ*=139.3 (d, *J*=6.6 Hz, Mes C_ipso_), 137.6 (d, *J*=5.2 Hz, Mes *C*CH_3_), 137.1 (d, *J*=5.9 Hz, Mes *C*CH_3_), 133.9 (d, *J*=9.8 Hz, Ph CH), 133.6 (d, *J*=9.3 Hz, Ph CH), 133.5 (d, *J*=3.8 Hz, Ar CH), 132.2 (d, *J*=2.9 Hz, Ar CH), 132.0 (d, ^1^ *J*=89.8 Hz, Ph C_ipso_), 131.5 (d, *J*=2.8 Hz, Ar CH), 130.2 (d, *J*=3.4 Hz, Ar CH), 130.0 (d, *J*=3.1 Hz, Ar CH), 129.8 (d, ^1^ *J*=90.8 Hz, Ph C_ipso_), 128.0, 127.7 (2×Ar CH), 119.9 (d, *J*=6.6 Hz, cyclopentadienyl C_ipso_N), 111.6 (cyclopentadienyl C_ipso_N), 72.4, 70.4 (d, *J*=9.8 Hz), 69.9, 68.1 (d, *J*=13.6 Hz), 67.8 (d, *J*=16.5 Hz), 67.5, 63.8 (7×cyclopentadienyl CH), 57.0 (d, ^1^ *J*=135.5 Hz, cyclopentadienyl C_ipso_P), 21.7 (d, *J*=1.6 Hz, Mes CH_3_), 21.1 (d, *J*=1.3 Hz, Mes CH_3_), 20.8 (d, *J*=1.5 Hz, Mes CH_3_), 3.3 ppm (two very closely spaced signals, 2×SiMe_3_). ^31^P{^1^H} NMR (202 MHz, C_6_D_6_): *δ*=23.3 ppm. ^119^Sn{^1^H} NMR (186 MHz, C_6_D_6_): *δ*=−1.3 ppm. Anal. calcd for C_37_H_46_N_3_FePSi_2_Sn (794.48): C 55.94, H 5.84, N 5.29 %; found C 55.97, H 6.16, N 5.03 %.

**Synthesis of 22**: A solution of mesityl azide (29 mg, 0.18 mmol) in *n*‐hexane (1 mL) was added to stirred solution of **14** (110 mg, 0.18 mmol) in *n*‐hexane (5 mL). Stirring was discontinued after 10 min. The solution was stored at −40 °C for crystallisation. The mother liquor was separated from the orange crystals, which were subsequently dried under reduced pressure. Yield 81 mg (67 %). ^1^H NMR (400 MHz, C_6_D_6_): *δ*=7.87–7.82, 7.40–7.35 (2 m, 2×2 H, Ph), 7.06 (m, 5 H, Ph and Mes CH), 6.88–6.82 (m, 3 H, Ph and Mes CH), 4.53, 4.13, 3.83, 3.81, 3.68, 3.65, 3.24 (7 m, 7×1 H, cyclopentadienyl H), 2.59 (s, 6 H, Mes *o*‐CH_3_), 2.36 (s, 3 H, Mes *p*‐CH_3_), 0.48, 0.33 ppm (2 s, 2×9 H, SiMe_3_). ^13^C{^1^H} NMR (101 MHz, C_6_D_6_): *δ*=153.5 (d, ^2^ *J*=6.6 Hz, Mes C_ipso_), 135.3 (d, *J*=10.7 Hz, Ar CH), 132.6 (d, *J*=10.3 Hz, Ar CH), 132.3 (d, ^4^ *J*=2.8 Hz, Ph *p*‐CH), 131.2 (d, ^1^ *J*=35.9 Hz, Ph C_ipso_), 131.0 (d, ^4^ *J*=2.8 Hz, Ph *p*‐CH), 129.3 (d, *J*=4.1 Hz, Ar CH), 129.3 (d, *J*=5.3 Hz, Ar CH), 129.0 (d, *J*=10.2 Hz, Ar CH), 127.1 (d, ^3^ *J*=9.7 Hz, Mes *o*‐*C*CH_3_), 126.6 (d, ^1^ *J*=43.6 Hz, phenyl C_ipso_), 122.0 (Mes *p*‐*C*CH_3_), 112.8 (d, *J*=20.7 Hz, cyclopentadienyl C_ipso_N), 102.0 (d, *J*=1.4 Hz, cyclopentadienyl C_ipso_N), 74.7 (d, *J*=2.3 Hz, cyclopentadienyl CH), 71.1 (cyclopentadienyl CH), 71.0 (d, *J*=7.0 Hz, cyclopentadienyl CH), 69.8 (cyclopentadienyl CH), 69.0 (d, *J*=4.2 Hz, cyclopentadienyl CH), 67.5 (cyclopentadienyl CH), 63.8 (d, *J*=1.4 Hz, cyclopentadienyl CH), 53.7 (d, ^1^ *J*=61.3 Hz, cyclopentadienyl C_ipso_P), 23.7 (d, ^4^ *J*=1.9 Hz, Mes *o‐*CH_3_), 23.1 (Mes *p*‐CH_3_), 2.7, 2.1 ppm (2×SiMe_3_). ^31^P{^1^H} NMR (202 MHz, C_6_D_6_): *δ*=−28.2 ppm. Anal. calcd for C_37_H_46_N_3_FeGePSi_2_ (748.40): C 59.38, H 6.20, N 5.61 %; found C 59.70, H 6.53, N 5.36 %.

**Synthesis of 23**: A solution of mesityl azide (29 mg, 0.18 mmol) in toluene (1 mL) was added to stirred solution of **14** (110 mg, 0.18 mmol) in toluene (5 mL). After 10 minutes the stirred solution was heated to 60 °C for 4 h. Volatile components were removed under reduced pressure. The orange residue was washed with a minimal amount of *n*‐hexane and was subsequently dried under reduced pressure. Yield 110 mg (83 %). ^1^H NMR (500 MHz, C_6_D_6_): *δ*=7.96–7.92 (m, 2 H, Ph *o*‐H), 7.64–7.59 (m, 2 H, Ph *o*‐H), 7.06–7.03 (m, 1 H, Ph *p*‐H), 6.96–6.85 (m, 5 H, Ph), 6.75, 6.67 (2 s, 2×1 H, Mes CH), 4.55, 4.37, 4.12, 4.10, 3.98, 3.75, 3.73 (7 m, 7×1 H, cyclopentadienyl H), 2.21 (s, 3 H, Mes CH_3_), 2.11 (d, *J*=2.3 Hz, 3 H, Mes CH_3_), 1.96 (d, *J*=1.8 Hz, 3 H, Mes CH_3_), 0.60, 0.38 ppm (2 s, 2×9 H, SiMe_3_). ^13^C{^1^H} NMR (101 MHz, C_6_D_6_): *δ*=139.0 (d, *J*=5.3 Hz, Mes C_ipso_), 138.2 (d, *J*=5.0 Hz, Mes *C*CH_3_), 138.1 (d, *J*=5.7 Hz, Mes *C*CH_3_), 133.9 (d, *J*=3.6 Hz, Mes *C*CH_3_), 133.7 (d, ^1^ *J*=90.3 Hz, Ph C_ipso_), 133.6 (d, *J*=9.8 Hz, Ph *o*‐CH), 133.0 (d, *J*=9.3 Hz, Ph *o‐*CH), 132.1 (d, *J*=2.9 Hz, Ph *p*‐CH), 131.5 (d, *J*=2.7 Hz, Ph *p*‐CH), 130.4 (d, *J*=3.1 Hz, Mes CH), 129.7 (d, *J*=2.8 Hz, Mes CH), 128.8 (second line of doublet concealed by solvent signal, Ph C_ipso_), 128.2, 127.9 (2×Ph *m*‐CH), 118.3 (d, *J*=5.7 Hz, cyclopentadienyl C_ipso_N), 109.6 (cyclopentadienyl C_ipso_N), 71.9 (cyclopentadienyl CH), 69.8 (d, *J*=9.0 Hz, cyclopentadienyl CH), 69.5 (cyclopentadienyl CH), 68.7 (d, *J*=13.5 Hz, cyclopentadienyl CH), 68.4 (cyclopentadienyl CH), 67.5 (d, *J*=14.9 Hz, cyclopentadienyl CH), 64.2 (cyclopentadienyl CH), 56.0 (d, ^1^ *J*=134.4 Hz, cyclopentadienyl C_ipso_P), 21.4 (d, *J*=1.2 Hz, Mes CH_3_), 21.3, 20.9 (2 d, *J*=1.5 Hz, 2×Mes CH_3_), 3.1, 3.0 ppm (2×SiMe_3_). ^31^P{^1^H} NMR (202 MHz, C_6_D_6_): *δ*=18.7 ppm. Anal. calcd for C_37_H_46_FeGeN_3_PSi_2_ (748.40): C 59.38, H 6.20, N 5.61 %; found C 60.31, H 6.58, N 5.52 %.

**Synthesis of 24**: CuCl (30 mg, 0.30 mmol) was added to a stirred solution of **17** (120 mg, 0.23 mmol) in toluene (5 mL). After 18 h insoluble material was removed by filtration through a short pad of celite, followed by washing with toluene (1.5 mL). Volatile components were removed from the combined filtrate and washing solution. The product was extracted from the residue with *n*‐hexane (3×2 mL). After filtration of the extract to remove trace amounts of insoluble material, the volume was reduced to ca. 0.5 mL. Yellow crystals were obtained after several days, which were separated from the mother liquor and subsequently dried under reduced pressure. Yield 61 mg (42 %). ^1^H NMR (400 MHz, C_6_D_6_): *δ*=3.84, 3.75 (2 m, 2×4 H, cyclopentadienyl H), 1.10 (s, 18 H, *t*Bu), 0.54 ppm (s, 12 H, SiMe_2_). ^13^C{^1^H} NMR (101 MHz, C_6_D_6_): *δ*=106.9 (C_ipso_), 69.3, 67.3 (2×cyclopentadienyl CH), 27.9 (C*Me*_3_), 19.2 (*C*Me_3_), −0.1 ppm (SiMe_2_). Anal. calcd for C_22_H_38_N_2_ClCuFeGeSi_2_ (614.19): C 43.02; H 6.24; N 4.56 %; found C 42.11; H 6.41; N 4.72 %.

**Synthesis of 25**: CuCl (25 mg, 0.21 mmol) was added to a stirred solution of **14** (90 mg, 0.15 mmol) in toluene (5 mL). After 72 h insoluble material was removed by filtration through a short pad of celite, followed by washing with toluene (1 mL). The volume of the combined filtrate and washing solution was reduced to ca. 0.5 mL. *n*‐Hexane (0.5 mL) was added. The solvent was slowly evaporated under ambient conditions, affording the crude product as a dark brownish red microcrystalline solid, which was washed with *n*‐hexane (2×0.2 mL) and subsequently dried under reduced pressure. Yield 25 mg (26 %). ^1^H NMR (400 MHz, C_6_D_6_): *δ*=7.95, 7.47 (2 br., 2×2 H, Ph), 7.31–7.29 (m, 5 H, Ph), 7.20–7.18 (m, 1 H, Ph), 7.10–7.05 (m, 4 H, Ph), 6.99–6.95 (m, 5 H, Ph), 6.87 (m, 1 H, Ph), 4.60, 4.22, 4.11, 3.98 (4 m, 4×1 H, cyclopentadienyl H), 3.89, 3.74 (2 m, 2×2 H, cyclopentadienyl H), 3.66–3.62 (m, 4 H, cyclopentadienyl H), 3.30, 3.02 (2 m, 2×1 H, cyclopentadienyl H), 0.53 (s, 18 H, SiMe_3_), 0.50, 0.24 ppm (2 s, 2×9 H, SiMe_3_). ^13^C{^1^H} NMR (101 MHz, C_6_D_6_): *δ*=135.7 (d, *J*=10.8 Hz), 134.4 (d, *J*=14.5 Hz), 134.2, 133.9, 133.7 (d, *J*=14.3 Hz), 132.7, 132.2, 132.1, 130.9, 129.9 (d, *J*=11.0 Hz), 129.7, 129.7, 129.4 (d, *J*=9.6 Hz), 128.6, 128.5, 128.2 (16×Ph), 109.3 (d, *J*=17.5 Hz), 105.1, 104.0 (3×cyclopentadienyl C_ipso_N, one of the expected four C_ipso_N signals and C_ipso_P signals not detected), 76.4 (d, *J*=6.6 Hz), 74.5, 72.4, 71.9 (d, *J*=5.7 Hz), 70.4, 69.3, 69.3, 68.3 (d, *J*=5.4 Hz), 67.5, 67.4, 66.7 (d, *J*=4.2 Hz), 66.3, 66.1, 65.1 (14×cyclopentadienyl CH), 5.1 (two isochronous signals, SiMe_3_), 3.6, 3.1 ppm (2×SiMe_3_). ^31^P{^1^H} NMR (202 MHz, C_6_D_6_): *δ*=−7.5, −17.5 ppm. Satisfactory microanalytical data could not be obtained.

**Synthesis of 26**: CuCl (10 mg, 0.10 mmol) was added to a stirred solution of **23** (67 mg, 0.09 mmol) in toluene (1 mL). After 24 h insoluble material was removed by filtration through a short pad of celite, followed by washing with toluene (1 mL). The volume of the combined filtrate and washing solution was reduced to ca. 0.5 mL. The solution was placed in a 5 mm NMR tube and was subsequently layered with *n*‐hexane (ca. 3 mL). After two weeks yellow crystals had formed, which were separated from the mother liquor and subsequently dried under reduced pressure. Yield 51 mg (67 %). ^1^H NMR (400 MHz, C_6_D_6_): *δ*=7.76–7.72, 7.54–7.49 (2 m, 2×2 H, Ph), 7.10–7.06 (m, 1 H, Ph), 6.93–6.89 (m, 3 H, Ph), 6.84 (s, 1 H, Mes CH), 6.83–6.79 (m, 2 H, Ph CH), 6.70 (s, 1 H, Mes CH), 4.43, 4.20, 4.05, 4.03, 3.82, 3.68, 3.65 (7 m, 7×1 H, cyclopentadienyl H), 2.11, 2.06, 1.97 (3 s, 3×3 H, Mes CH_3_), 0.62, 0.41 ppm (2 s, 2×9 H, SiMe_3_). ^13^C{^1^H} NMR (101 MHz, C_6_D_6_): *δ*=138.4 (d, *J*=4.0 Hz), 138.0 (d, *J*=4.7 Hz), 136.7 (d, *J*=3.0 Hz), 136.1 (d, *J*=4.8 Hz, Mes C_ipso_), 133.6 (d, *J*=10.2 Hz, Ph *m*‐CH), 133.1 (d, *J*=2.8 Hz), 132.8 (d, *J*=9.4 Hz, Ph *m*‐CH), 132.6 (d, *J*=2.8 Hz), 131.8 (d, *J*=2.6 Hz), 131.5 (d, ^1^ *J*=93.8 Hz, Ph C_ipso_), 130.3 (d, *J*=2.5 Hz), 128.5, 128.4 (2×Ph *o*‐CH), 125.3 (d, ^1^ *J*=96.1 Hz, Ph C_ipso_), 113.5 (d, *J*=6.0 Hz, cyclopentadienyl C_ipso_N), 105.6 (cyclopentadienyl C_ipso_N), 73.0 (cyclopentadienyl CH), 71.1 (d, *J*=9.5 Hz, cyclopentadienyl CH), 70.3 (d, *J*=12.6 Hz, cyclopentadienyl CH), 69.9, 69.6 (2×cyclopentadienyl CH), 68.0 (d, *J*=14.3 Hz, cyclopentadienyl CH), 65.8 (cyclopentadienyl CH), 54.7 (d, ^1^ *J*=126.6 Hz, cyclopentadienyl C_ipso_P), 22.2 (d, *J*=1.1 Hz, Mes CH_3_), 21.2 (d, *J*=1.3 Hz, Mes CH_3_), 20.9 (d, *J*=1.2 Hz, Mes CH_3_), 3.9, 3.6 ppm (2×SiMe_3_). ^31^P{^1^H} NMR (202 MHz, C_6_D_6_): *δ*=26.0 ppm. Anal. calcd for C_37_H_46_ClCuFeGeN_3_PSi_2_ (847.40): C 52.44, H 5.47, N 4.96 %; found C 53.28, H 4.47, N 5.54 %.

**X‐ray crystallography**: For each data collection a single crystal was mounted on a micro‐mount and all geometric and intensity data were taken from this sample at 100(2) K, except for **23**, which was measured at 253(2) K due to a phase transition below this temperature. Data collections were carried out either on a Stoe IPDS2 diffractometer equipped with a 2‐circle goniometer and an area detector on a Stoe StadiVari diffractometer equipped with a 4‐circle goniometer and a DECTRIS Pilatus 200 K detector. The data sets were corrected for absorption, Lorentz and polarisation effects. The structures were solved by direct methods (SHELXT) and refined using alternating cycles of least‐squares refinements against *F* ^2^ (SHELXL2014/7).^[62]^ C‐bonded H atoms were included in the models in calculated positions, heteroatom‐bonded H atoms have been found in the difference Fourier lists. All H atoms were treated with the 1.2‐fold or 1.5‐fold isotropic displacement parameter of their bonding partner. Experimental details for each diffraction experiment are given in Table S1 in the Supporting Information.

CCDC 2159886 (for **17**), 2159887 (for **18**), 2159888 (for **19**), 2159889 (for **20**⋅Et_2_O), 2159890 (for **21**), 2159891 (for **22**), 2159892 (for **23**), 2159893 (for **24**), 2159894 (for **26**), 2159895 (for **25**) contain the supplementary crystallographic data for this paper. These data are provided free of charge by the joint Cambridge Crystallographic Data Centre and Fachinformationszentrum Karlsruhe Access Structures service.

## Conflict of interest

The authors declare no conflict of interest.

1

## Supporting information

As a service to our authors and readers, this journal provides supporting information supplied by the authors. Such materials are peer reviewed and may be re‐organized for online delivery, but are not copy‐edited or typeset. Technical support issues arising from supporting information (other than missing files) should be addressed to the authors.

Supporting Information

## Data Availability

The data that support the findings of this study are available in the supplementary material of this article.
