# Supplementary material for: Reactions of Mesityl Azide with Ferrocene‐Based N‐Heterocyclic Germylenes, Stannylenes and Plumbylenes, Including PPh2‐Functionalised Congeners
Source: Chemistry. 2022 Jun 8;28(42):e202200996. doi: 10.1002/chem.202200996 (PMC9400874; doi:10.1002/chem.202200996)
Supplement: Supplementary file 1 — Supporting Information [file CHEM-28-0-s001.pdf]

Chemistry–A European Journal

Supporting Information

Reactions of Mesityl Azide with Ferrocene-Based N-Heterocyclic Germylenes, Stannylenes and Plumbylenes, Including PPh_2-Functionalised Congeners

Robin Guthardt, Lisa Oetzel, Tobias Lang, Clemens Bruhn, and Ulrich Siemeling*

Table of Contents

**A**	**X-Ray Crystallography**	S2
**B**	**Plots of NMR Spectra**	S4

A X-Ray Crystallography

Table S1. X-ray crystallographic details.

	**17**	**18**	**19**	**20**·Et_2O	**21**
Empirical formula	$C_{22}H_{38}FeGeN_2Si_2$	$C_{40}H_{60}FeN_6Si_2Sn$	$C_{31}H_{49}FeGeN_3Si_2$	$C_{78}H_{101}Fe_2N_{10}OP_2Pb_2Si_4$	$C_{37}H_{46}FeN_3PSi_2Sn$
Formula weight	515.16	855.66	648.35	1895.06	794.46
Crystal system	monoclinic	monoclinic	triclinic	triclinic	triclinic
Space group	$P2_1/n$	$P2_1/c$	P-1	P-1	P-1
a/Å	7.2876(2)	17.9932(5)	13.2543(8)	12.3497(8)	10.6278(4)
b/Å	10.9098(3)	19.3689(7)	15.9696(9)	13.0361(12)	12.9582(5)
c/Å	31.6151(9)	24.7472(8)	18.3777(11)	13.6017(12)	13.5657(5)
α/°	90	90	64.139(4)	99.119(7)	90.506(3)
β/°	92.228(2)	103.420(2)	71.524(5)	109.871(6)	96.071(3)
γ/°	90	90	74.894(5)	94.152(6)	105.090(3)
Volume/Å^3	2511.70(12)	8389.1(5)	3285.6(4)	2014.5(3)	1792.41(12)
Z	4	8	4	1	2
ρ_{calcd}/gcm^{-3}	1.362	1.355	1.311	1.562	1.472
μ/mm^{-1}	7.083	8.317	5.535	12.130	10.075
F(000)	1080.0	3568.0	1368.0	949.0	816.0
Crystal size/mm^3	0.29 × 0.20 × 0.11	0.20 × 0.09 × 0.01	0.18 × 0.10 × 0.05	0.11 × 0.08 × 0.03	0.18 × 0.11 × 0.05
Radiation used	Cu Kα (λ = 1.54186 Å)	Cu Kα (λ = 1.54186 Å)	Cu Kα (λ = 1.54186 Å)	Cu Kα (λ = 1.54186 Å)	Cu Kα (λ = 1.54186 Å)
2Θ range/°	8.574 to 142.056	5.05 to 142.226	6.516 to 143.036	6.932 to 143.268	6.558 to 141.938
	−4 ≤ h ≤ 8	−21 ≤ h ≤ 10	−6 ≤ h ≤ 15	−15 ≤ h ≤ 13	−12 ≤ h ≤ 12
Index ranges	−12 ≤ k ≤ 13	−19 ≤ k ≤ 23	−18 ≤ k ≤ 19	−16 ≤ k ≤ 8	−15 ≤ k ≤ 12
	−32 ≤ l ≤ 38	−27 ≤ l ≤ 30	−20 ≤ l ≤ 22	−13 ≤ l ≤ 16	−16 ≤ l ≤ 11
Reflections collected	16531	34153	25187	17183	14258
Independent reflections	4741 [R_{int} = 0.0212]	15585 [R_{int} = 0.0651]	12187 [R_{int} = 0.0727]	7516 [R_{int} = 0.0203]	6652 [R_{int} = 0.0245]
Data/restraints/parameters	4741/0/263	15585/0/933	12187/0/740	7516/0/477	6652/0/415
Goodness-of-fit on F^2	1.027	1.029	1.028	1.077	1.065
Final R indexes	R_1 = 0.0325	R_1 = 0.0730	R_1 = 0.0667	R_1 = 0.0278	R_1 = 0.0376
[$I > 2\sigma(I)$]	wR_2 = 0.0874	wR_2 = 0.1941	wR_2 = 0.1318	wR_2 = 0.0711	wR_2 = 0.0995
Final R indexes	R_1 = 0.0337	R_1 = 0.0908	R_1 = 0.1296	R_1 = 0.0303	R_1 = 0.0388
[all data]	wR_2 = 0.0886	wR_2 = 0.2122	wR_2 = 0.1589	wR_2 = 0.0727	wR_2 = 0.1006
Largest diff. peak/hole/eÅ^{-3}	0.30/−0.67	3.17/−1.39	0.70/−0.96	0.91/−1.54	0.93/−1.34
CCDC No.	2159886	2159887	2159888	2159889	2159890

Table S1 (continued). X-ray crystallographic details.

	**22**	**23**	**24**	**25**	**26**
Empirical formula	$C_{37}H_{46}FeGeN_3PSi_2$	$C_{37}H_{46}FeGeN_3PSi_2$	$C_{88}H_{152}Cl_4Cu_4Fe_4Ge_4N_8Si_8$	$C_{56}H_{70}ClCuFe_2Ge_2N_4P_2Si_4$	$C_{37}H_{46}ClCuFeGeN_3PSi_2$
Formula weight	748.36	748.36	2456.61	1329.33	847.35
Crystal system	monoclinic	triclinic	triclinic	monoclinic	monoclinic
Space group	$P2_1/c$	P-1	P-1	$P2_1/c$	$P2_1/n$
a/Å	10.0569(3)	10.6099(3)	12.3055(9)	14.4545(7)	19.4471(7)
b/Å	20.2949(6)	13.2540(4)	16.0214(10)	14.1563(8)	19.1033(5)
c/Å	18.5061(6)	13.4675(4)	29.489(2)	34.2639(15)	22.1141(8)
α/°	90	89.845(3)	89.107(6)	90	90
β/°	104.287(3)	95.211(3)	89.804(6)	97.527(4)	106.980(3)
γ/°	90	104.423(3)	71.582(5)	90	90
Volume/Å^3	3660.3(2)	1826.19(10)	5515.2(7)	6950.7(6)	7857.3(5)
Z	4	2	2	4	8
ρ_{calcd}/gcm^{-3}	1.358	1.361	1.479	1.270	1.433
μ/mm^{-1}	5.451	1.359	2.562	6.302	1.860
F(000)	1560.0	780.0	2528.0	2728.0	3488.0
Crystal size/mm^3	0.38 × 0.18 × 0.04	0.27 × 0.21 × 0.11	0.41 × 0.2 × 0.03	0.15 × 0.08 × 0.01	0.25 × 0.20 × 0.13
Radiation used	Cu Kα (λ = 1.54186 Å)	Mo Kα (λ = 0.71073 Å)	Mo Kα (λ = 0.71073 Å)	Cu Kα (λ = 1.54186 Å)	Mo Kα (λ = 0.71073 Å)
2Θ range/°	6.578 to 143.33	4.348 to 65.182	3.488 to 62.692	8.13 to 138.998	2.458 to 51.632
Index ranges	−5 ≤ h ≤ 11<br>−24 ≤ k ≤ 24<br>−22 ≤ l ≤ 20	−14 ≤ h ≤ 15<br>−20 ≤ k ≤ 19<br>−20 ≤ l ≤ 20	−17 ≤ h ≤ 16<br>−23 ≤ k ≤ 14<br>−41 ≤ l ≤ 43	−8 ≤ h ≤ 17<br>−17 ≤ k ≤ 15<br>−41 ≤ l ≤ 34	−23 ≤ h ≤ 19<br>−23 ≤ k ≤ 20<br>−27 ≤ l ≤ 26
Reflections collected	17106	32668	59642	25342	33912
Independent reflections	6787 [R_{int} = 0.0162]	11902 [R_{int} = 0.0444]	30637 [R_{int} = 0.0600]	12232 [R_{int} = 0.1019]	14911 [R_{int} = 0.0413]
Data/restraints/parameters	6787/0/415	11902/0/415	30637/12/1219	12232/123/716	14911/0/874
Goodness-of-fit on F^2	1.028	1.079	0.964	1.022	1.015
Final R indexes [$I > 2\sigma(I)$]	R_1 = 0.0277<br>wR_2 = 0.0668	R_1 = 0.0748<br>wR_2 = 0.2040	R_1 = 0.0694<br>wR_2 = 0.1636	R_1 = 0.0908<br>wR_2 = 0.2248	R_1 = 0.0441<br>wR_2 = 0.0906
Final R indexes [all data]	R_1 = 0.0350<br>wR_2 = 0.0709	R_1 = 0.0996<br>wR_2 = 0.2478	R_1 = 0.1366<br>wR_2 = 0.1985	R_1 = 0.1703<br>wR_2 = 0.2854	R_1 = 0.0731<br>wR_2 = 0.1006
Largest diff. peak/hole/eÅ^{-3}	0.29/–0.29	1.99/–1.45	1.59/–1.14	0.87/–0.96	0.63/–0.49
CCDC No.	2159891	2159892	2159893	2159895	2159894

B Plots of NMR Spectra

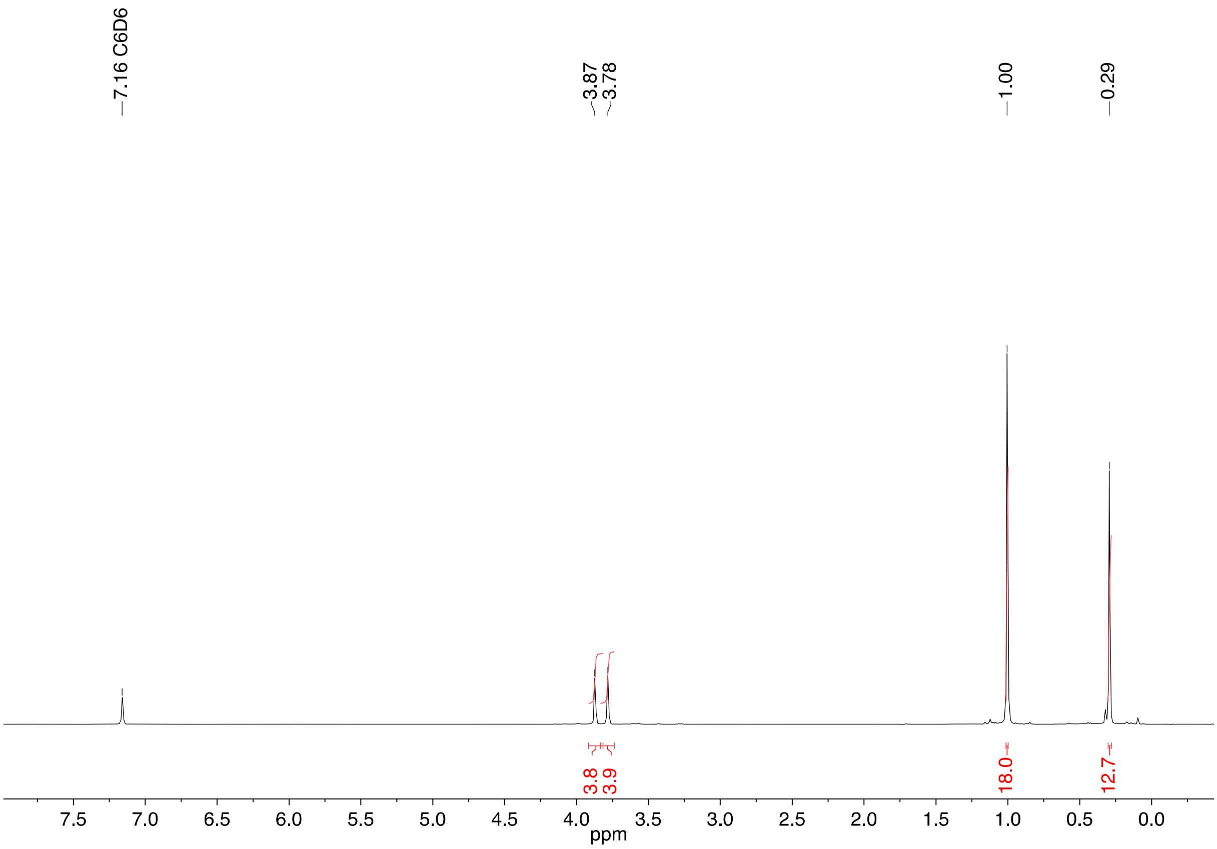

Figure S1. ^{1}H NMR spectrum (400 MHz, C_6D_6) of **17**.

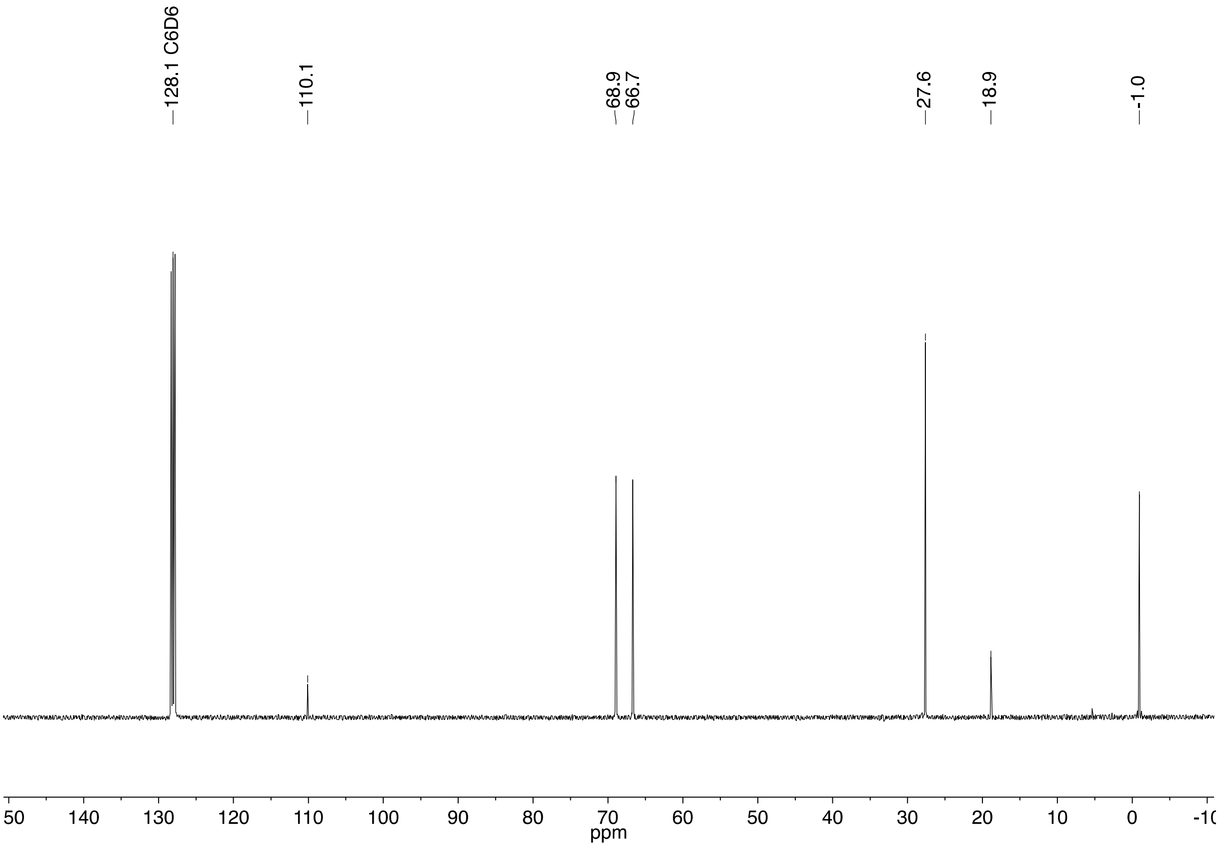

Figure S2. $^{13}C\{^{1}H\}$ NMR spectrum (101 MHz, C_6D_6) of **17**.

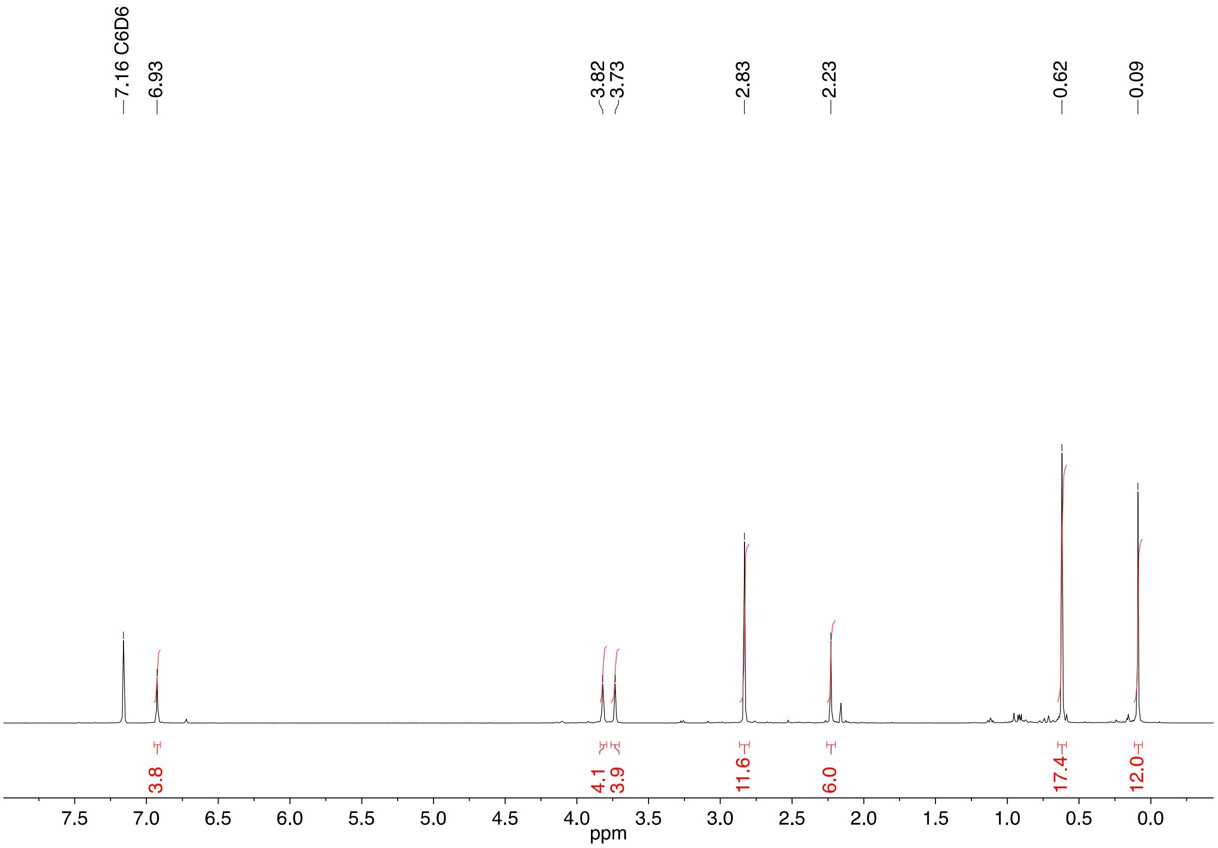

Figure S3. ^{1}H NMR spectrum (400 MHz, C_6D_6) of **18**.

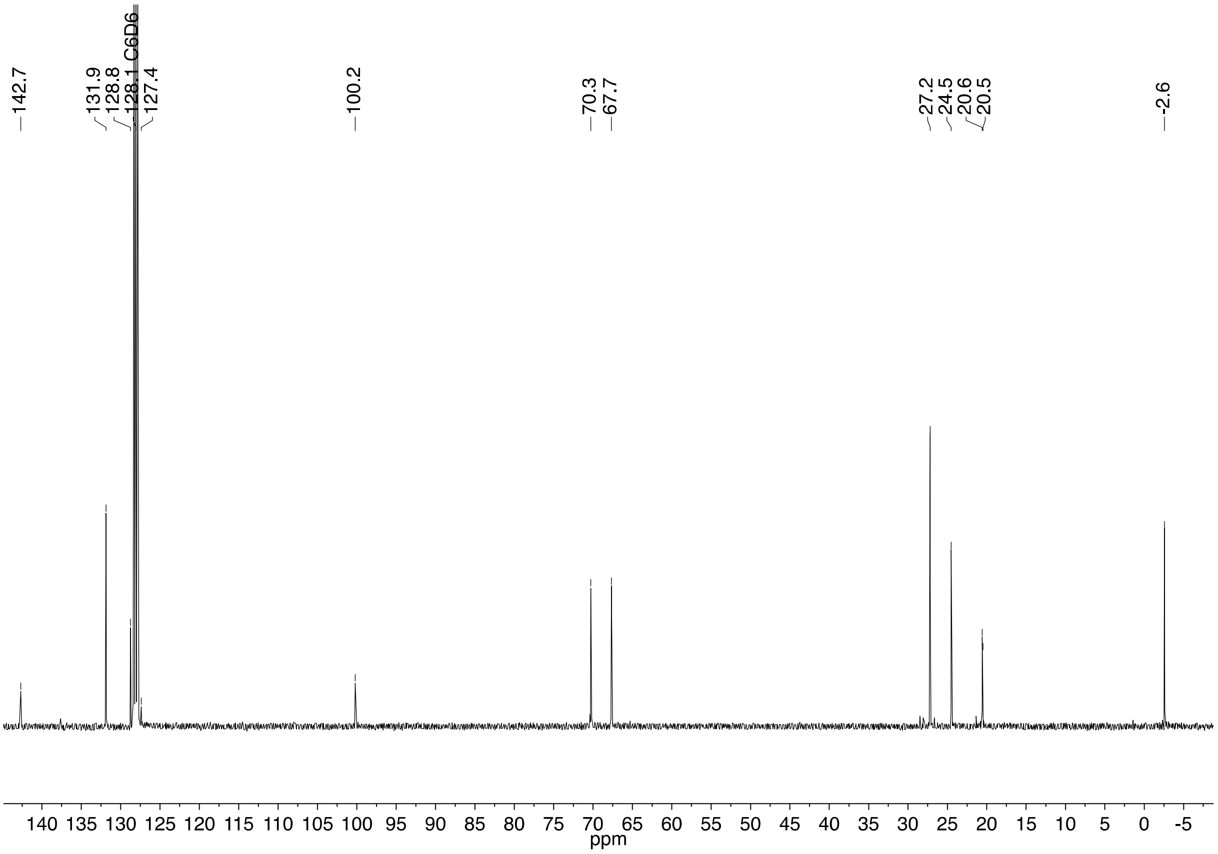

Figure S4. $^{13}C\{^{1}H\}$ NMR spectrum (101 MHz, C_6D_6) of **18**.

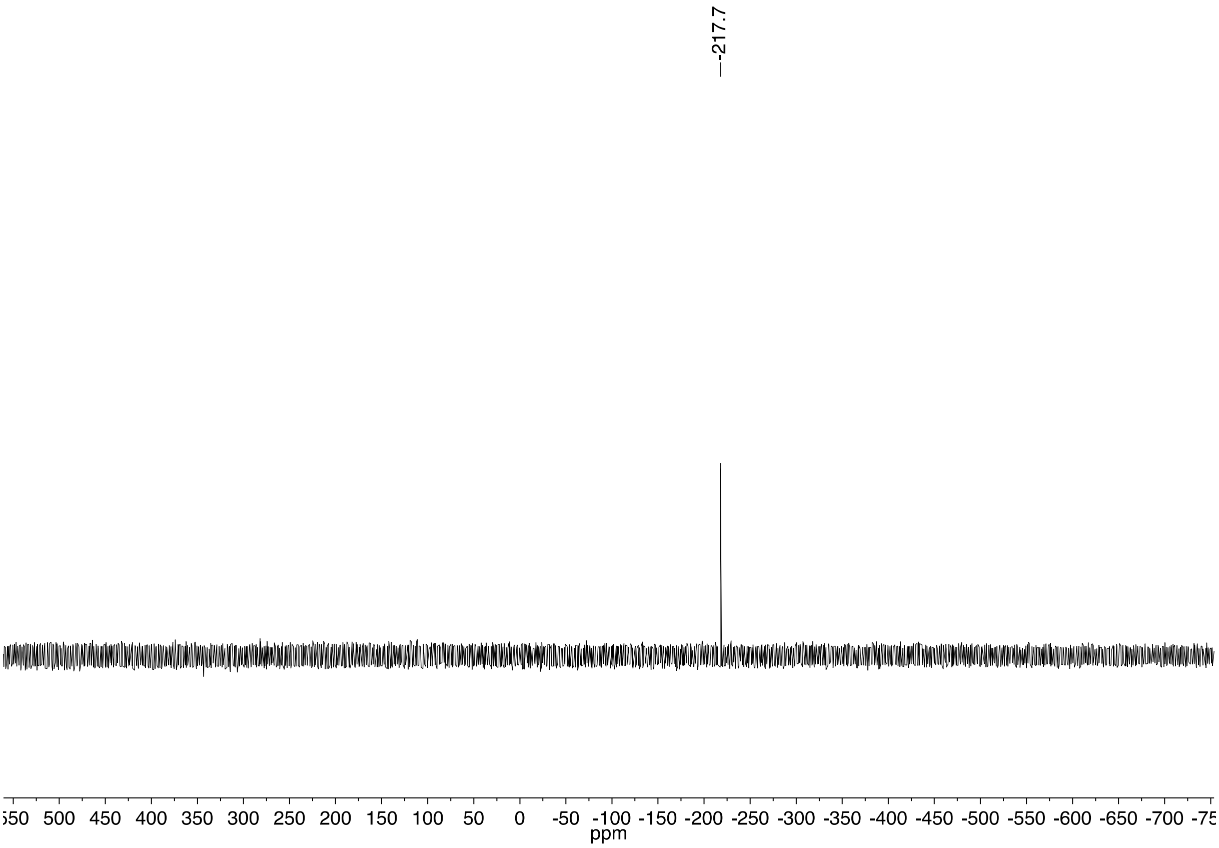

Figure S5. $^{119}Sn\{^{1}H\}$ NMR spectrum (186 MHz, C_6D_6) of **18**.

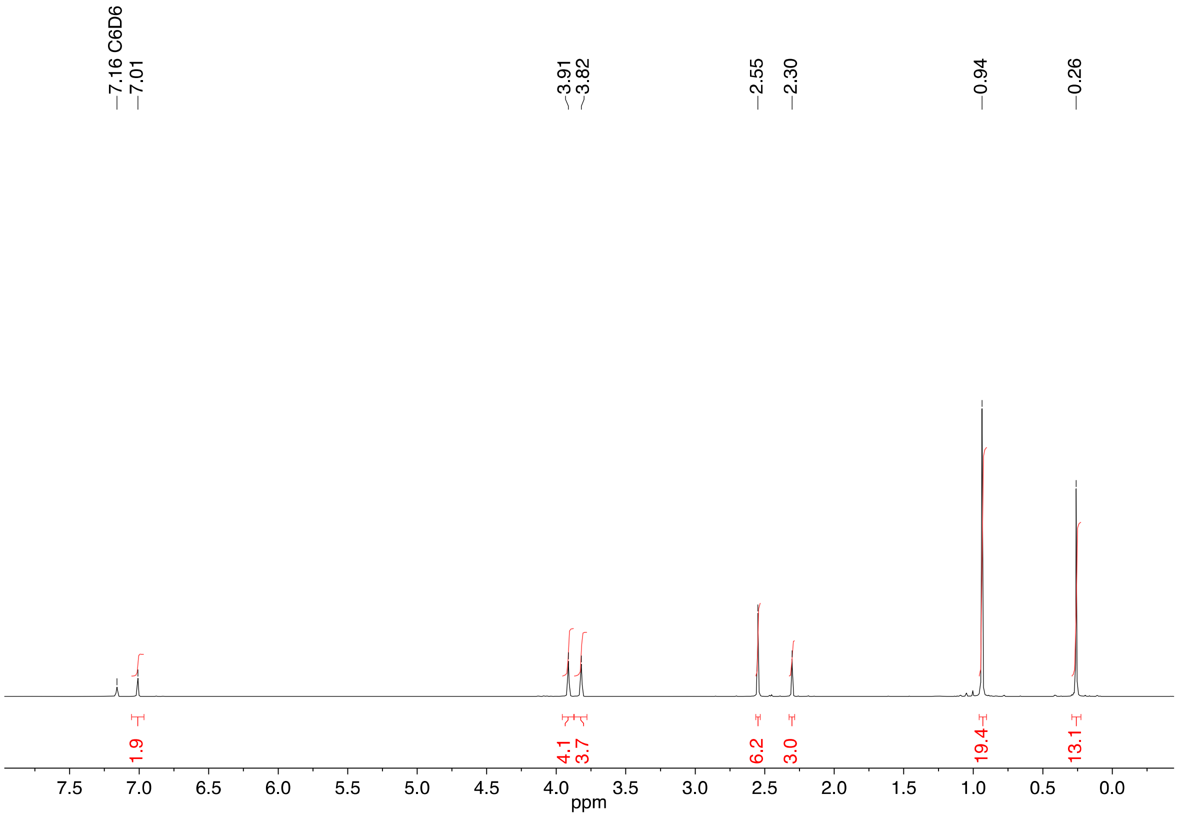

Figure S6. ^{1}H NMR spectrum (400 MHz, C_6D_6) of **19**.

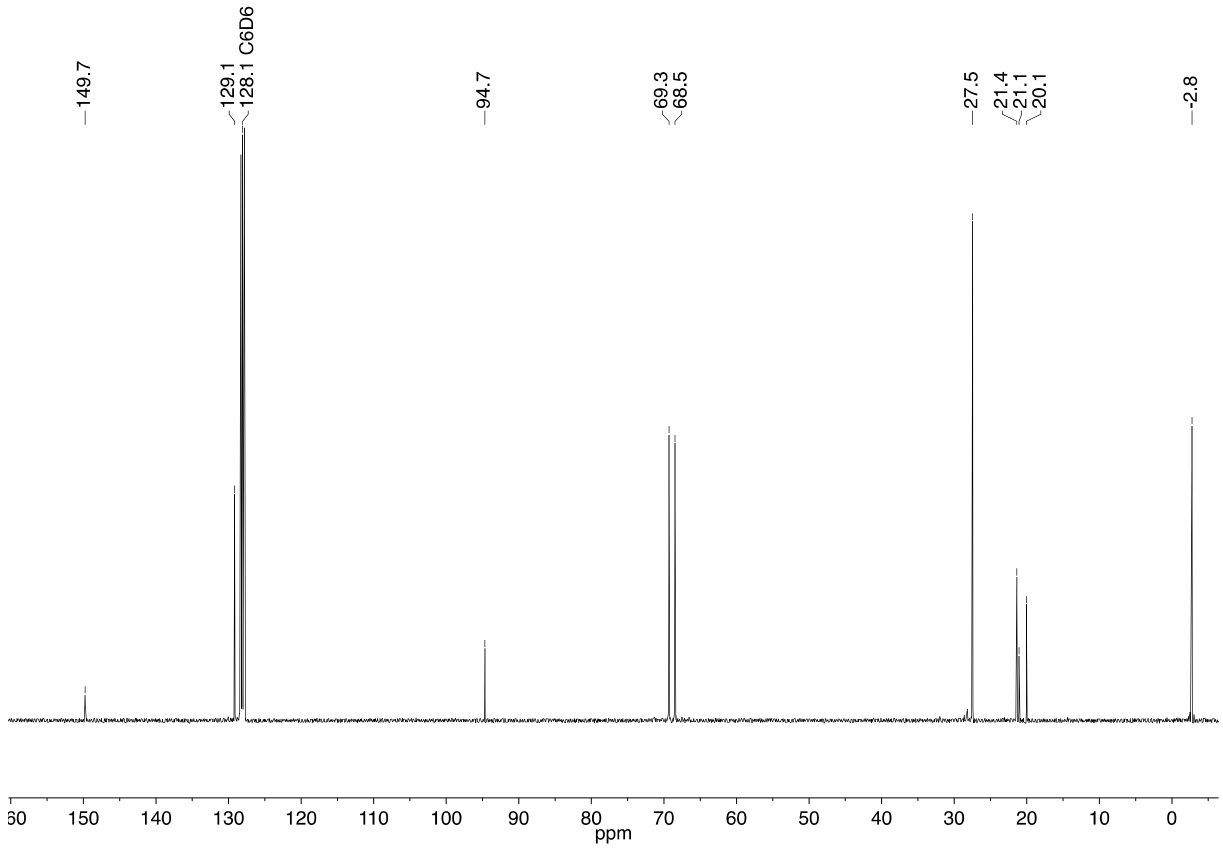

Figure S7. $^{13}C\{^{1}H\}$ NMR spectrum (101 MHz, C_6D_6) of **19**.

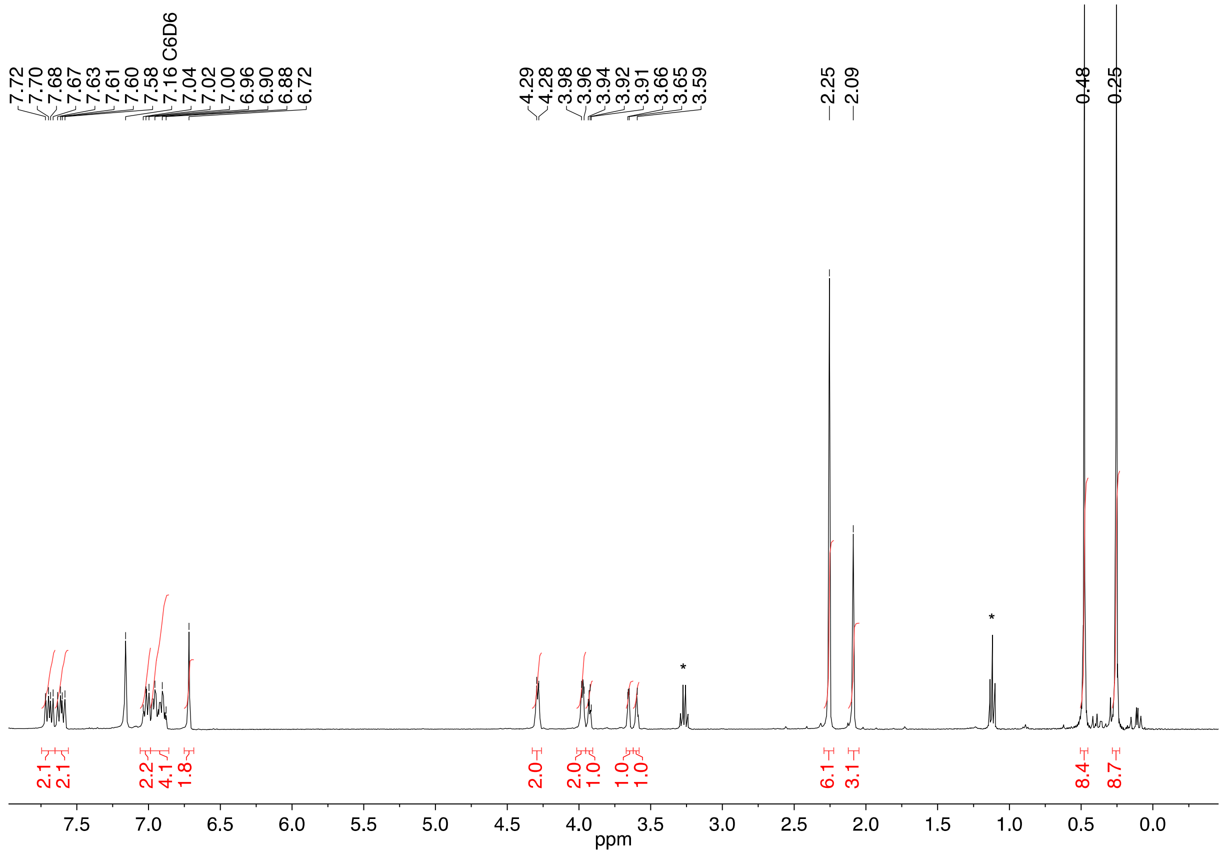

Figure S8. ^{1}H NMR spectrum (400 MHz, C_6D_6) of **20**. Signals marked (*) belong to residual diethyl ether.

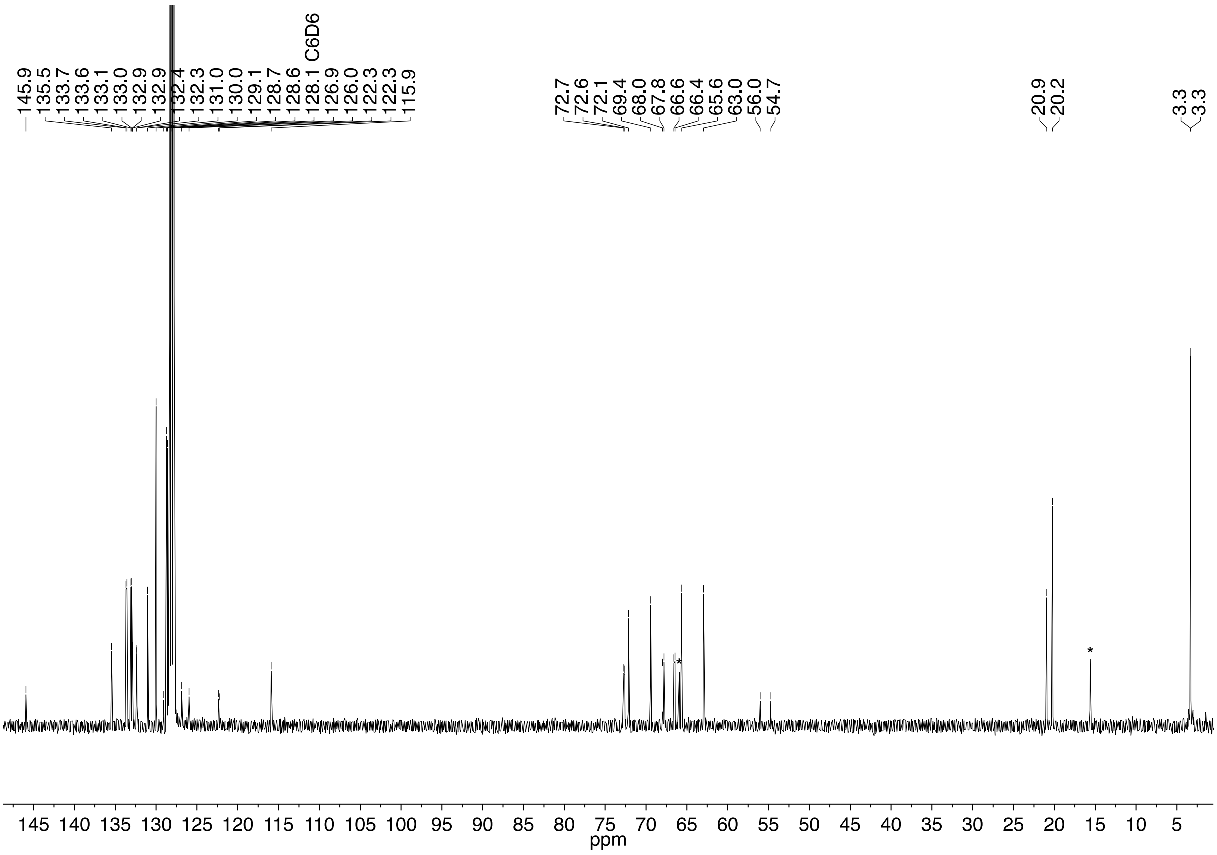

Figure S9. $^{13}C\{^{1}H\}$ NMR spectrum (101 MHz, C_6D_6) of **20**. Signals marked (*) belong to residual diethyl ether.

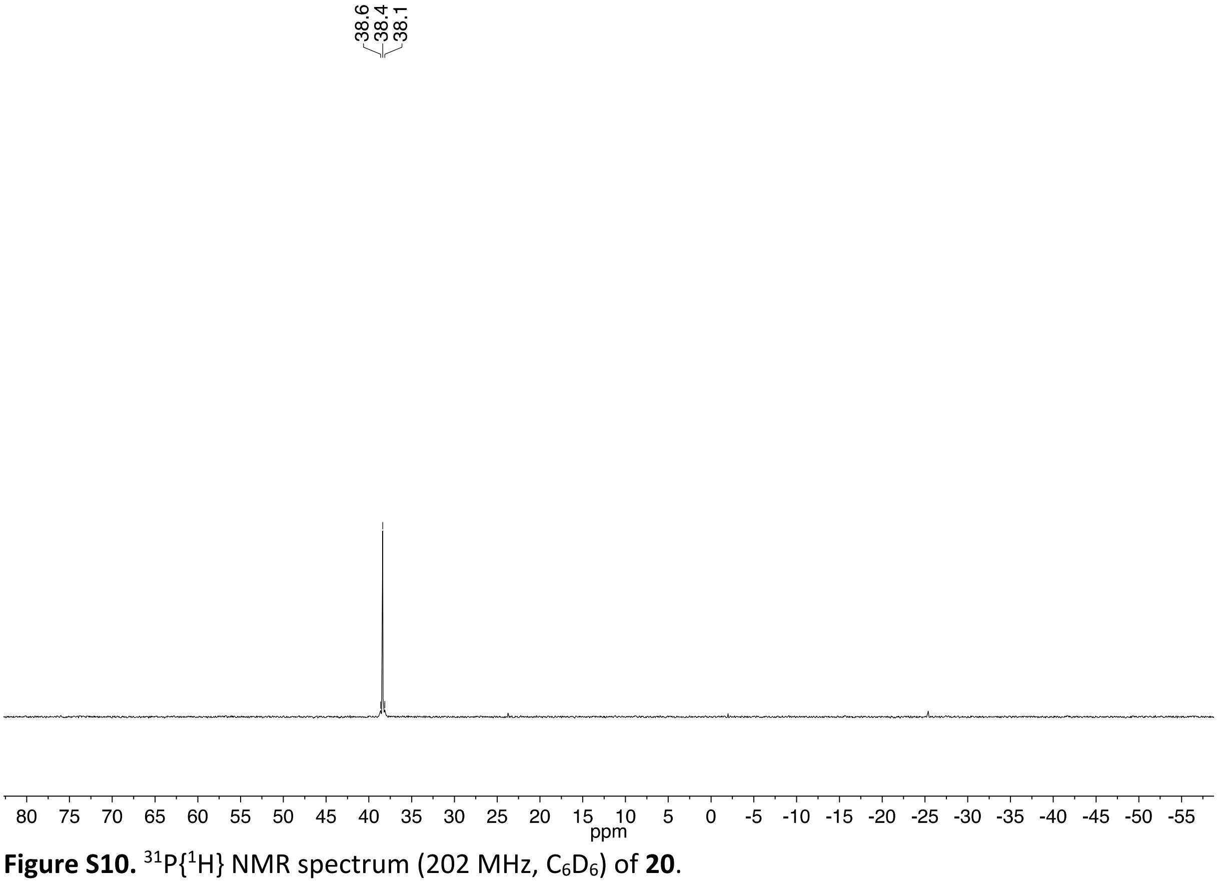

Figure S10. $^{31}P\{^{1}H\}$ NMR spectrum (202 MHz, C_6D_6) of **20**.

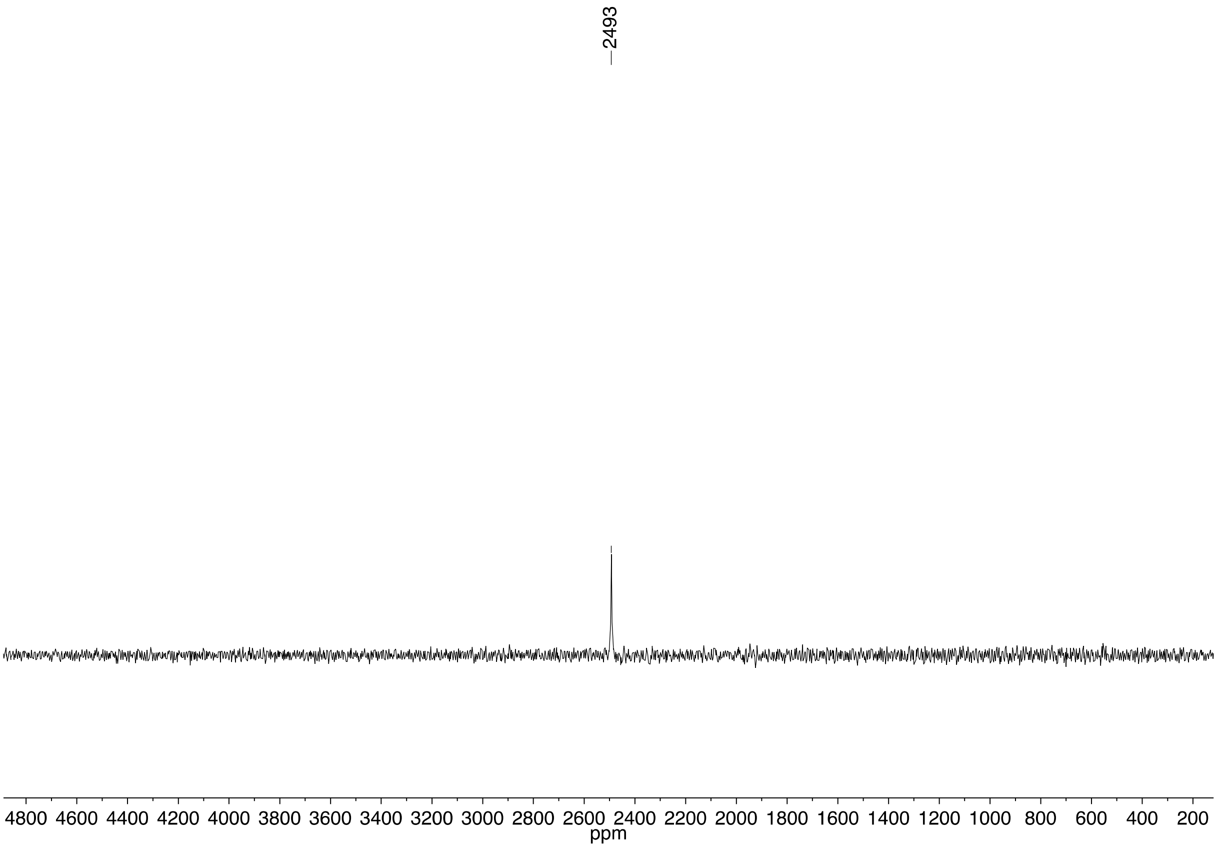

Figure S11. ^{207}Pb NMR spectrum (105 MHz, C_6D_6) of **20**.

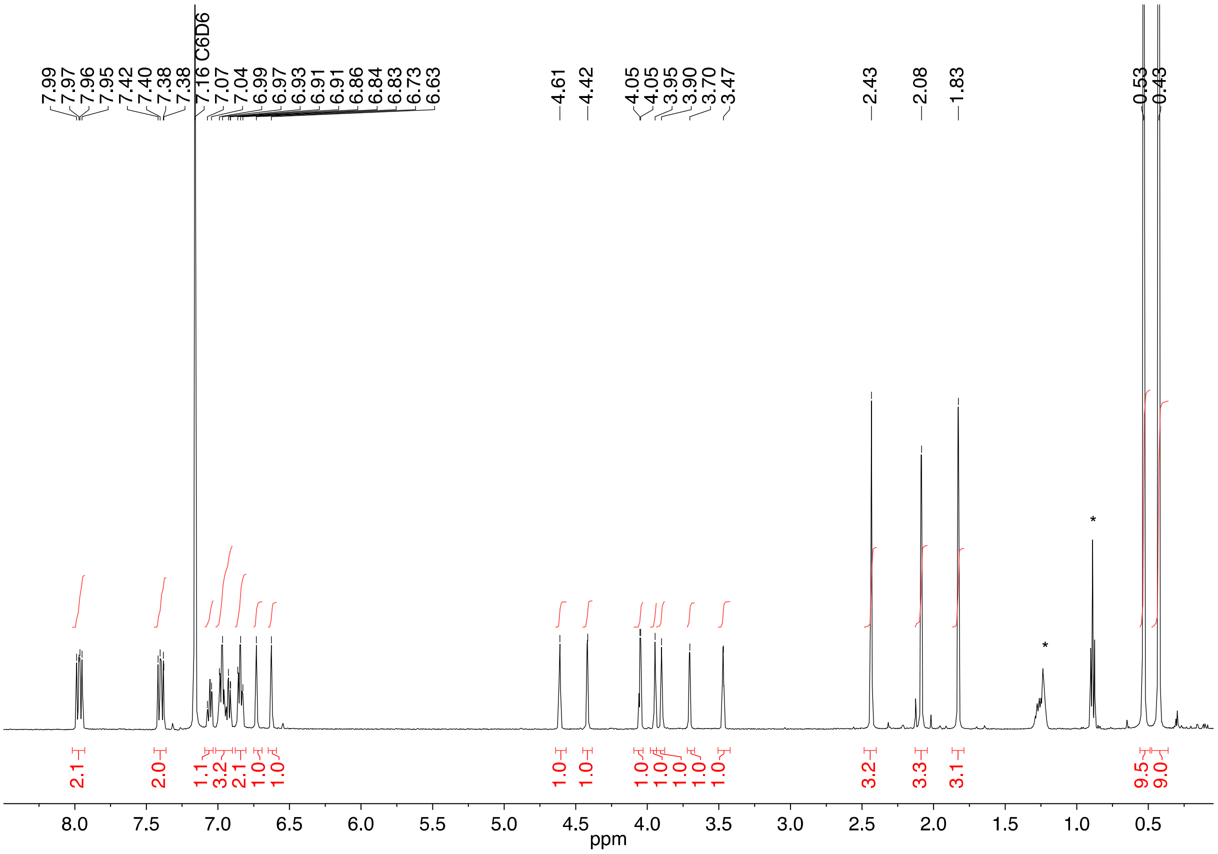

Figure S12. ^{1}H NMR spectrum (500 MHz, C_6D_6) of **21**. Signals marked (*) belong to residual *n*-hexane.

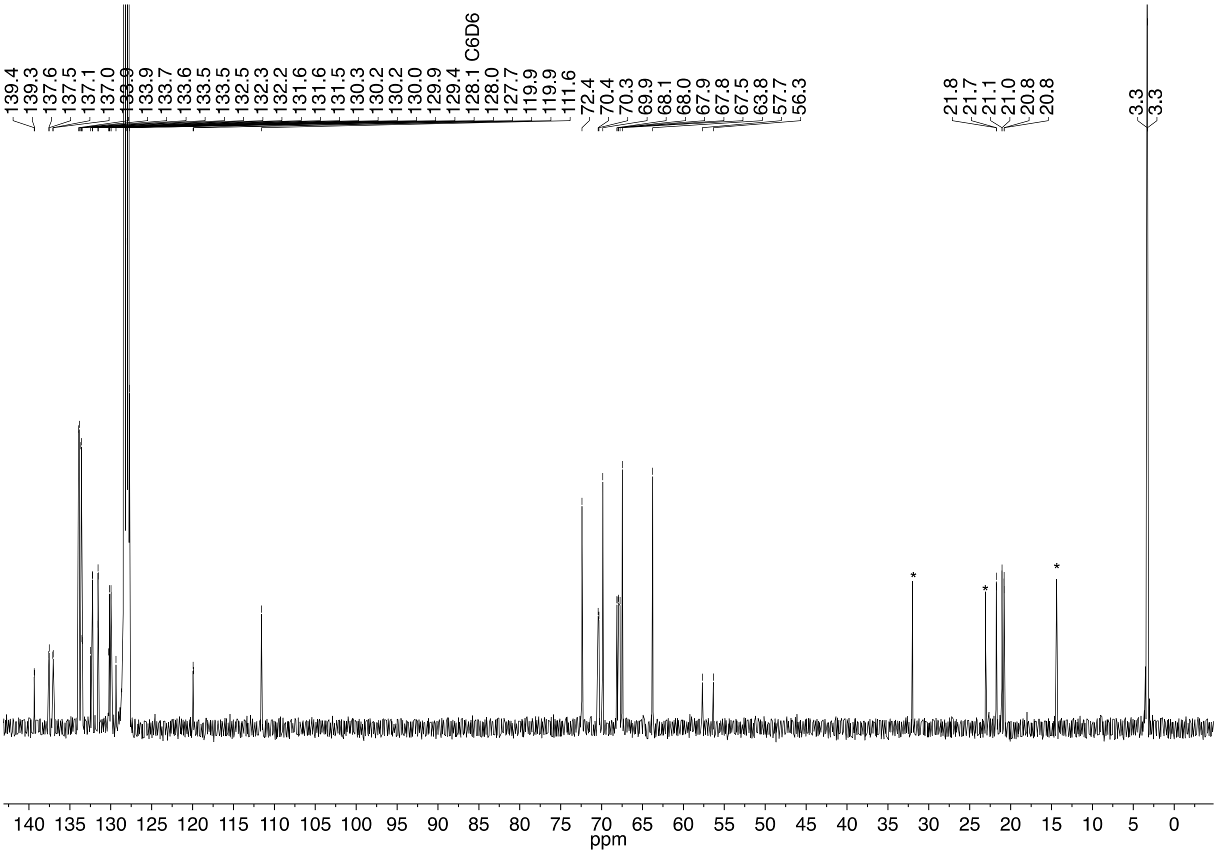

Figure S13. $^{13}C\{^1H\}$ NMR spectrum (101 MHz, C_6D_6) of **21**. Signals marked (*) belong to residual *n*-hexane.

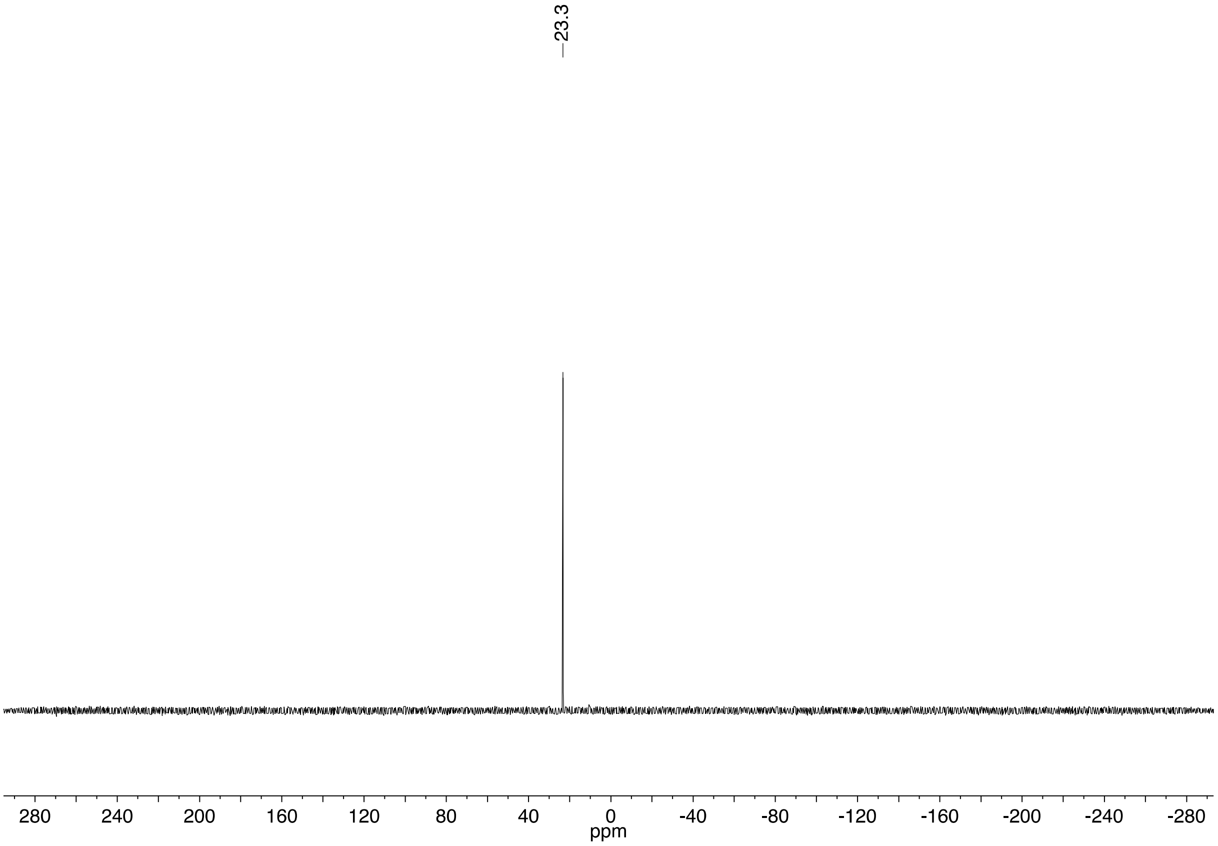

Figure S14. $^{31}P\{^1H\}$ NMR spectrum (202 MHz, C_6D_6) of **21**.

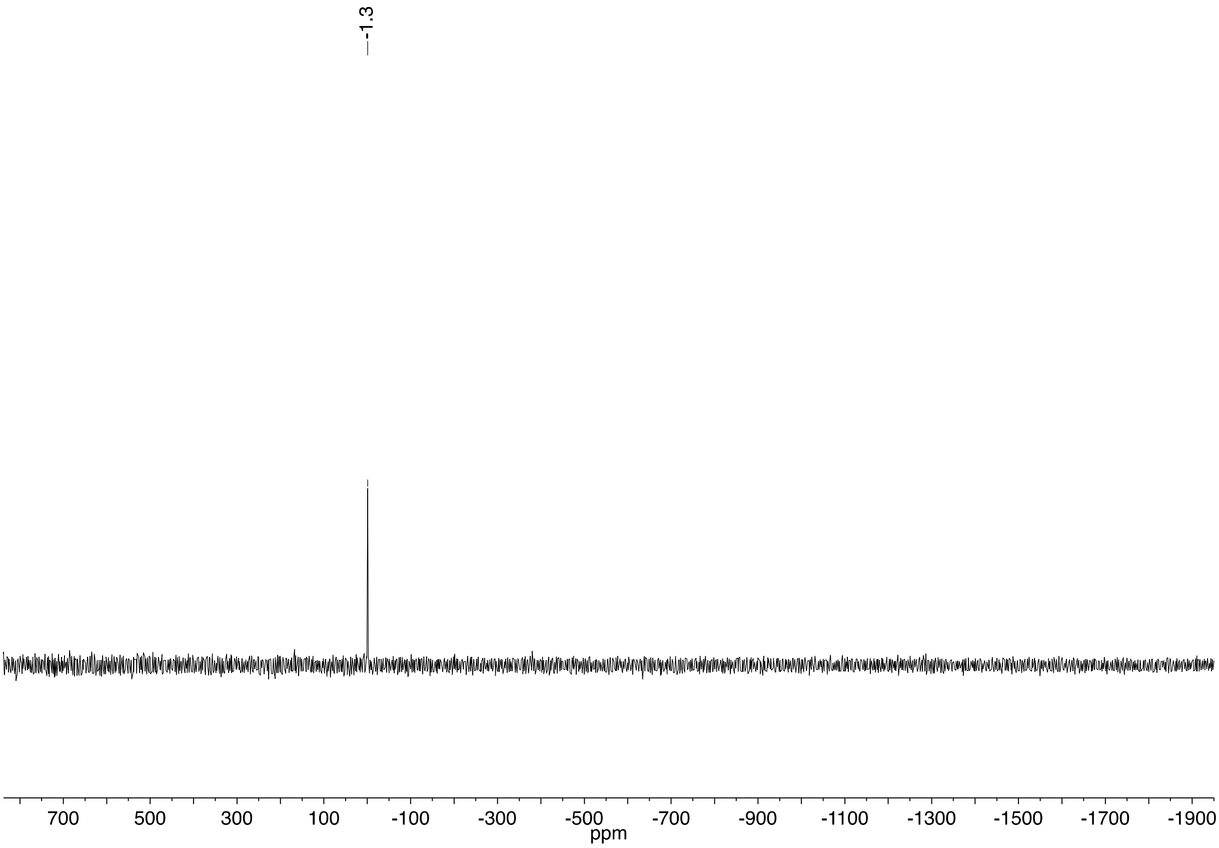

Figure S15. $^{119}Sn\{^{1}H\}$ NMR spectrum (186 MHz, C_6D_6) of **21**.

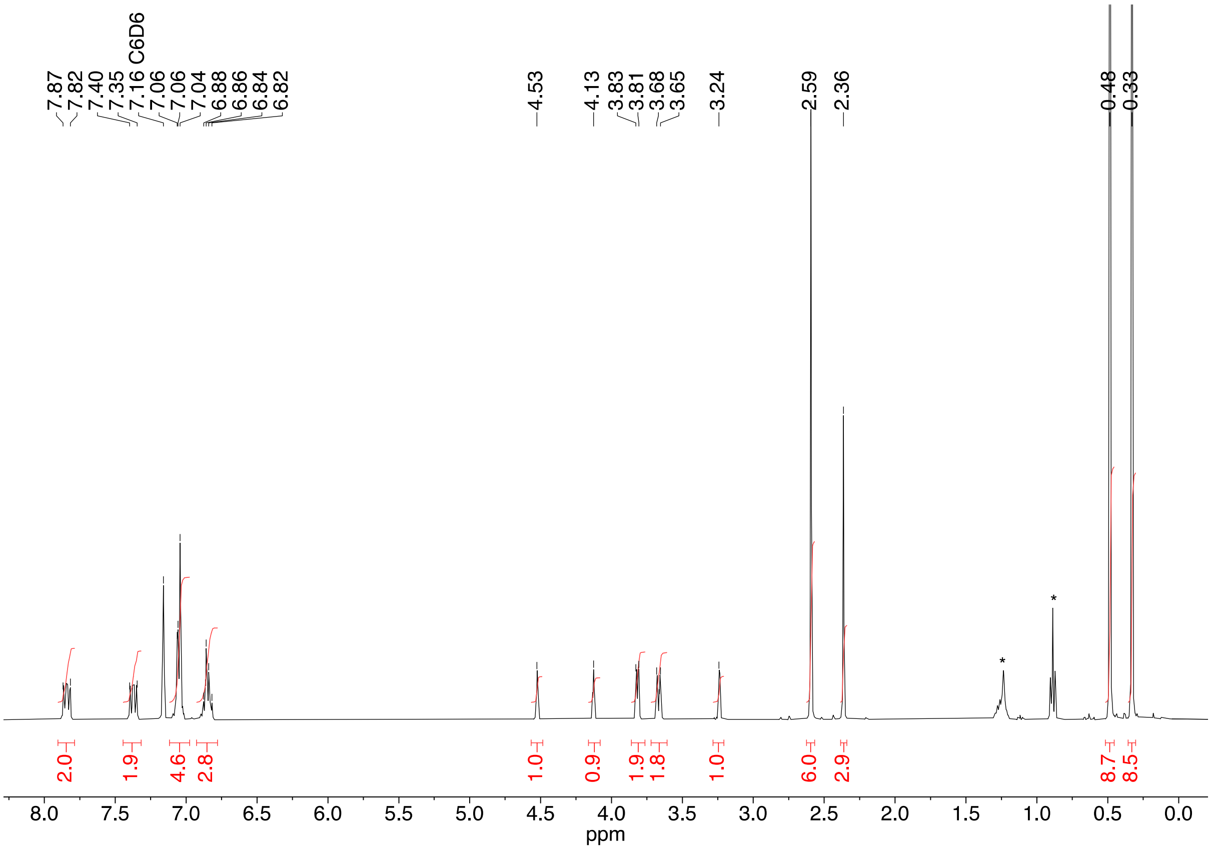

Figure S16. ^{1}H NMR spectrum (400 MHz, C_6D_6) of **22**. Signals marked (*) belong to residual *n*-hexane.

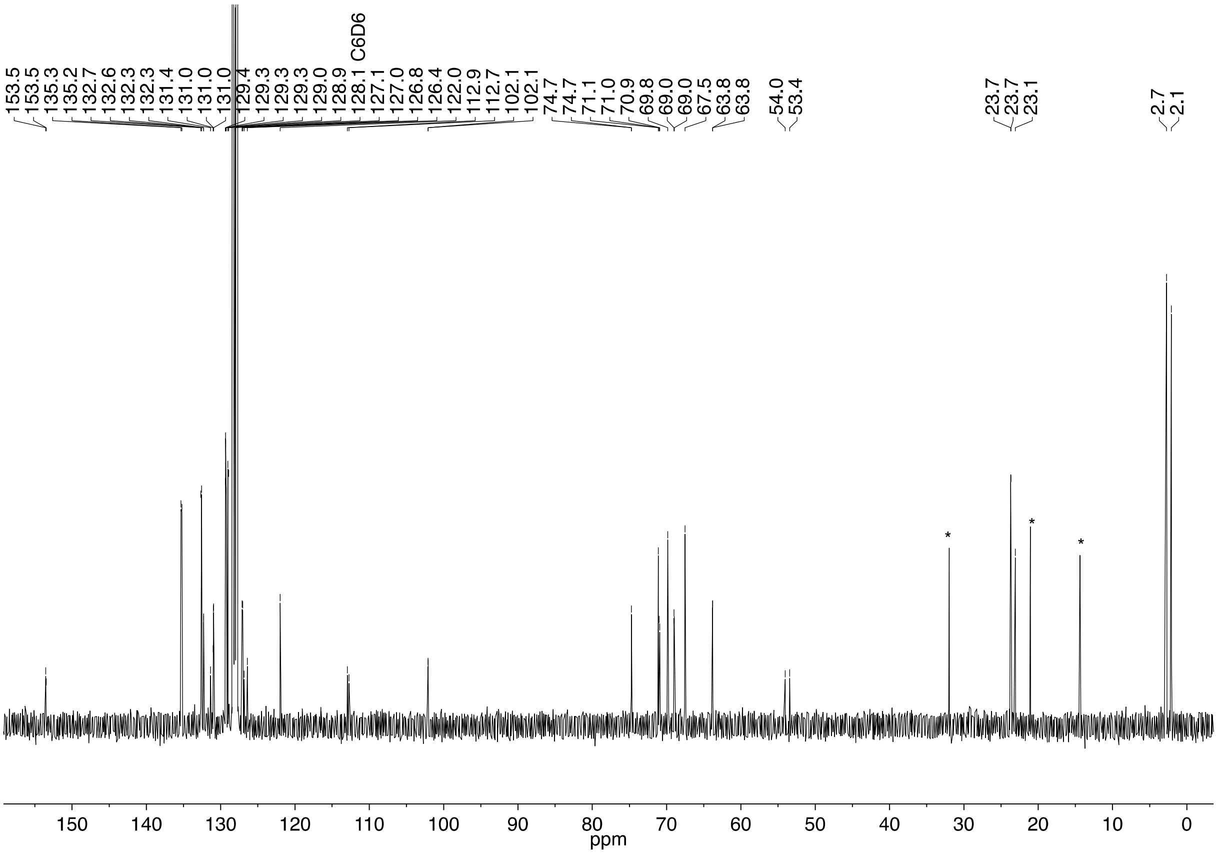

Figure S17. $^{13}C\{^{1}H\}$ NMR spectrum (101 MHz, C_6D_6) of **22**. Signals marked (*) belong to residual *n*-hexane.

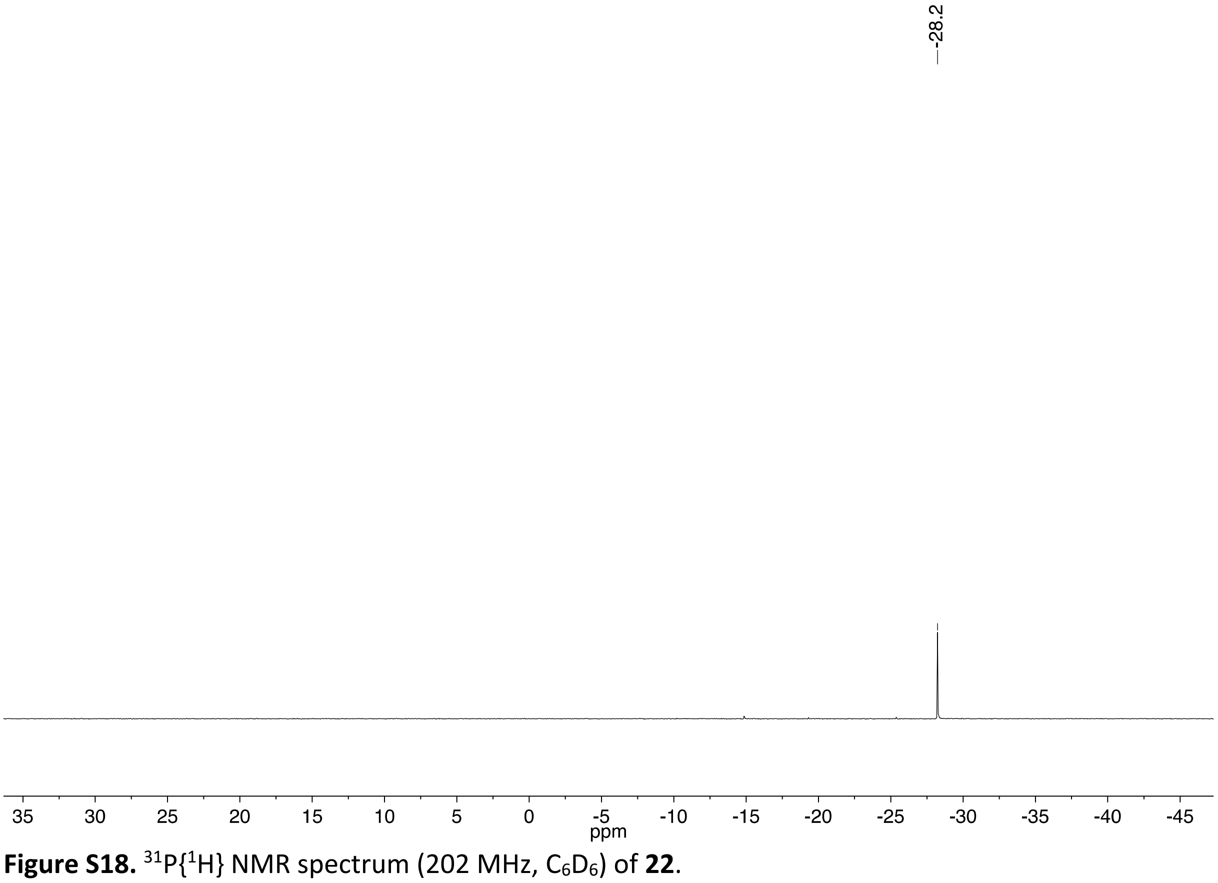

Figure S18. $^{31}P\{^{1}H\}$ NMR spectrum (202 MHz, C_6D_6) of **22**.

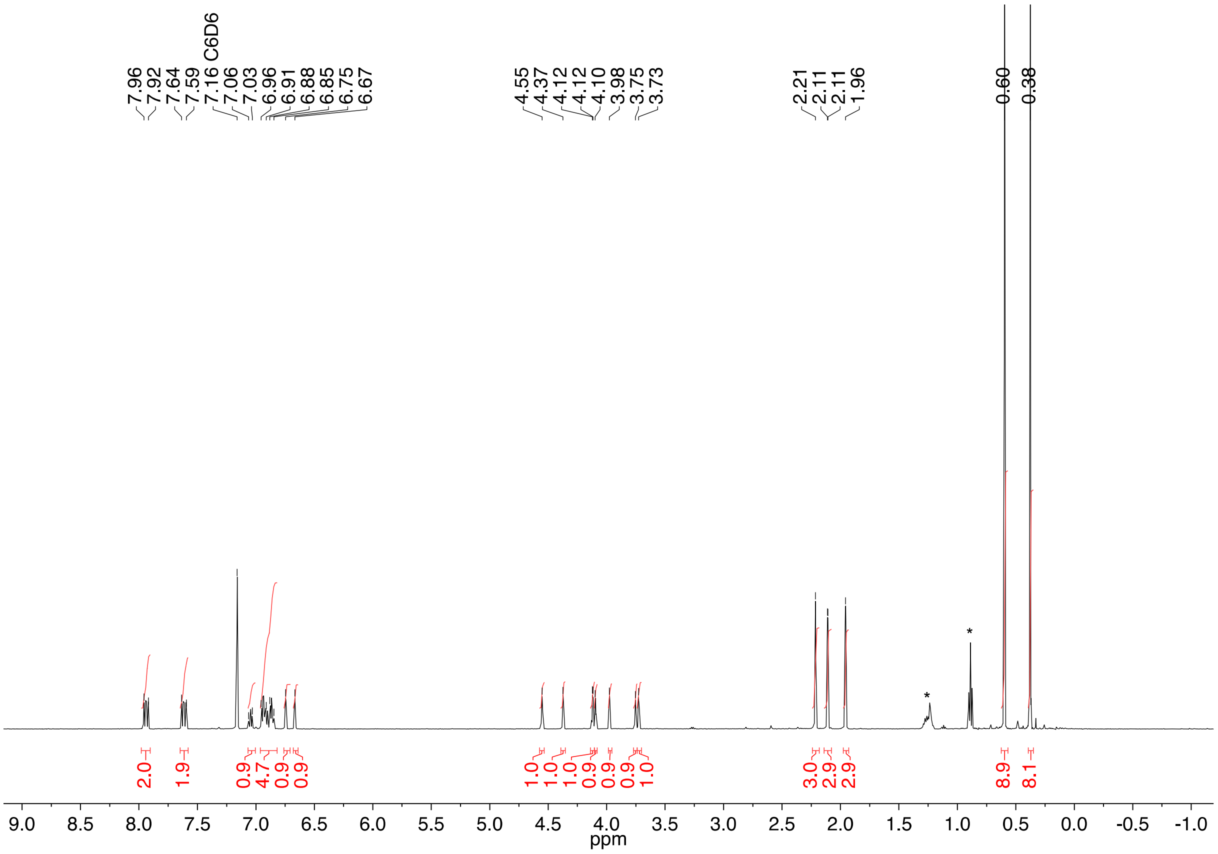

Figure S19. ^{1}H NMR spectrum (400 MHz, C_6D_6) of **23**. Signals marked (*) belong to residual *n*-hexane.

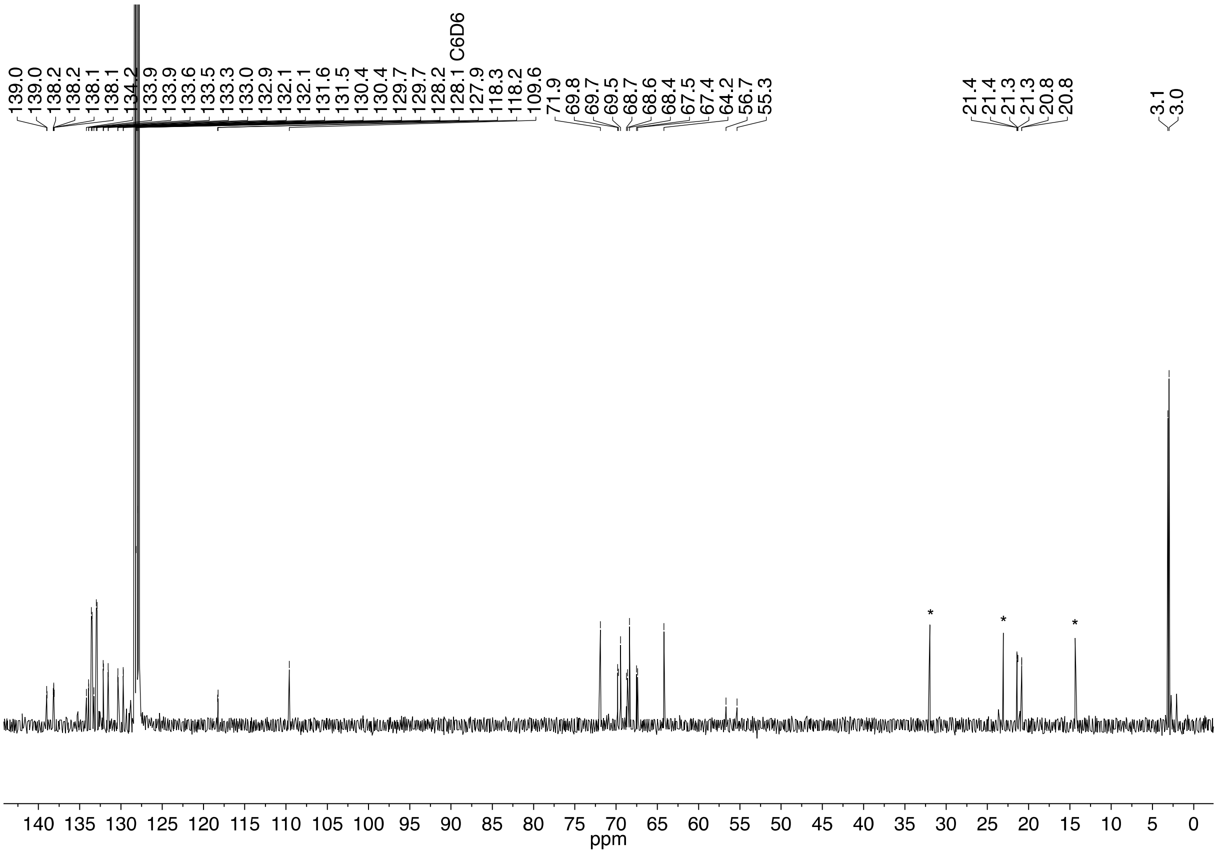

Figure S20. $^{13}C\{^{1}H\}$ NMR spectrum (101 MHz, C_6D_6) of **23**. Signals marked (*) belong to residual *n*-hexane.

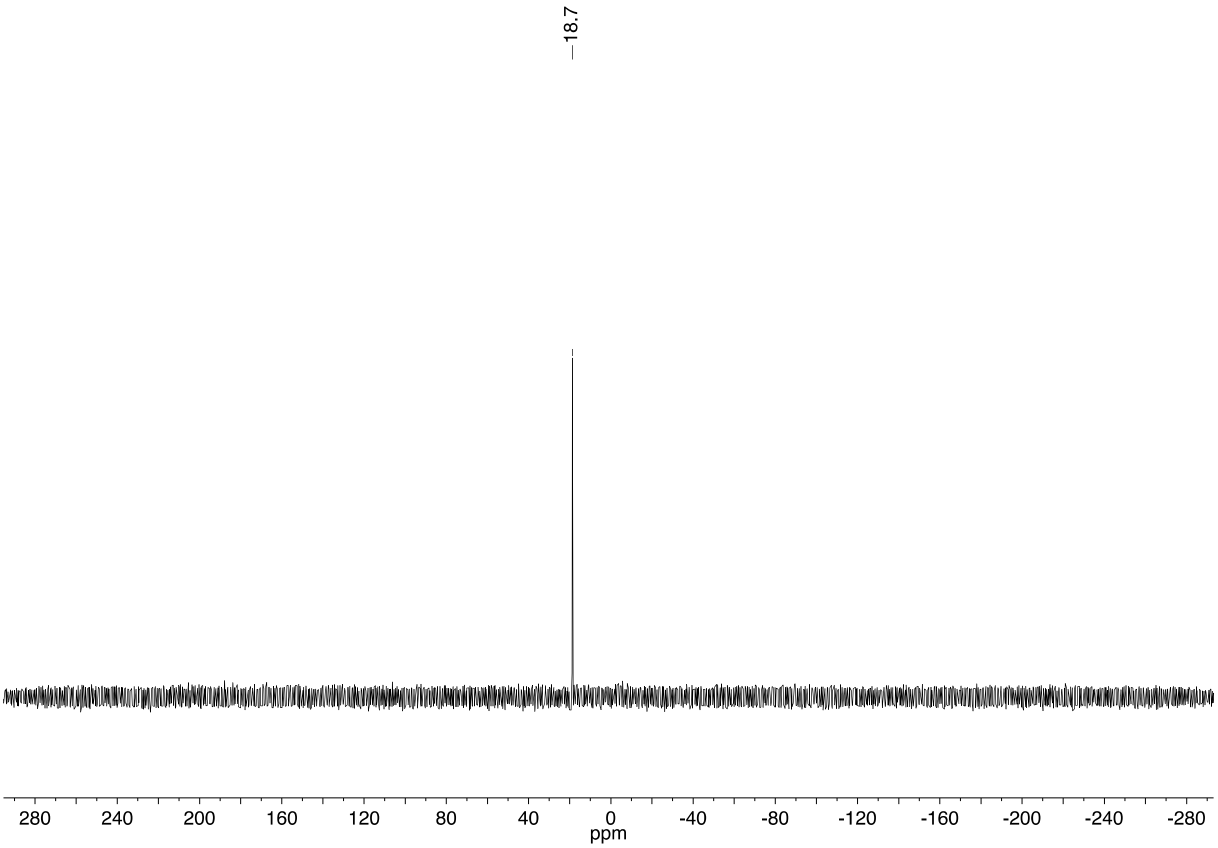

Figure S21. $^{31}P\{^{1}H\}$ NMR spectrum (202 MHz, C_6D_6) of **23**.

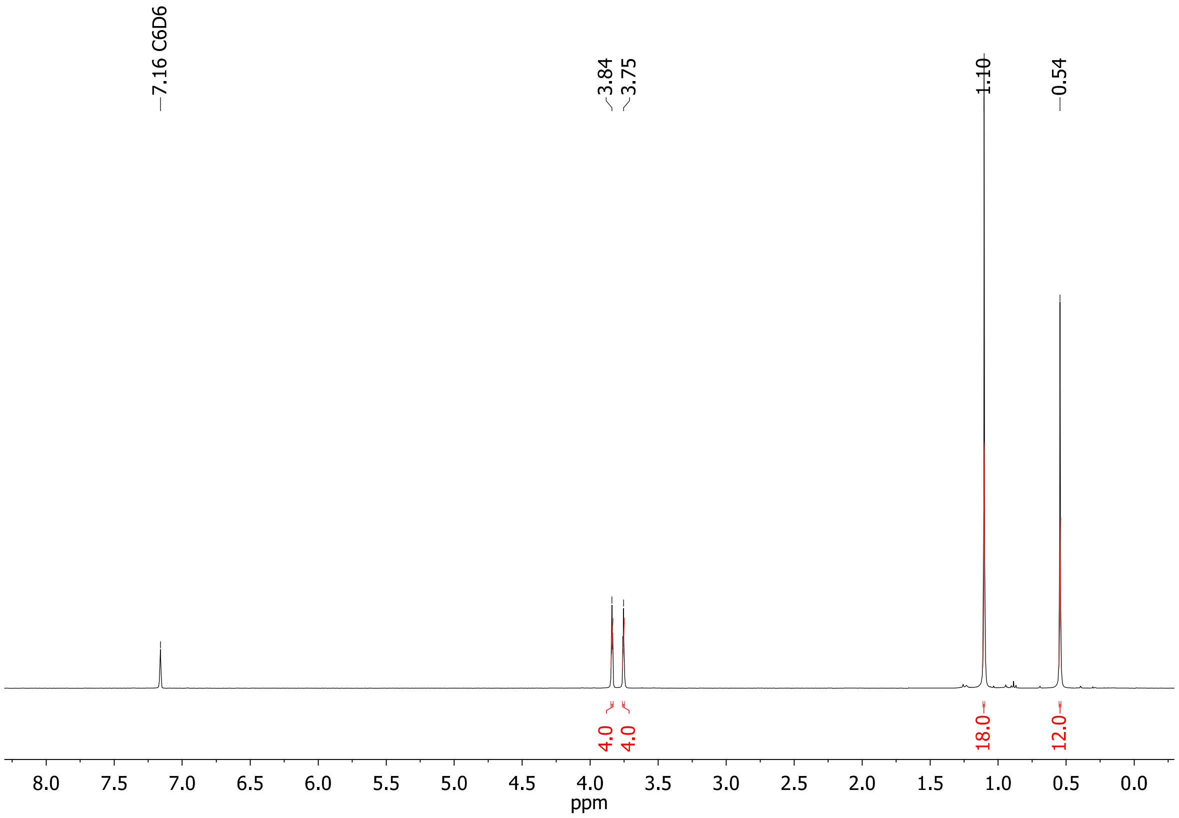

Figure S22. ^{1}H NMR spectrum (400 MHz, C_6D_6) of **24**.

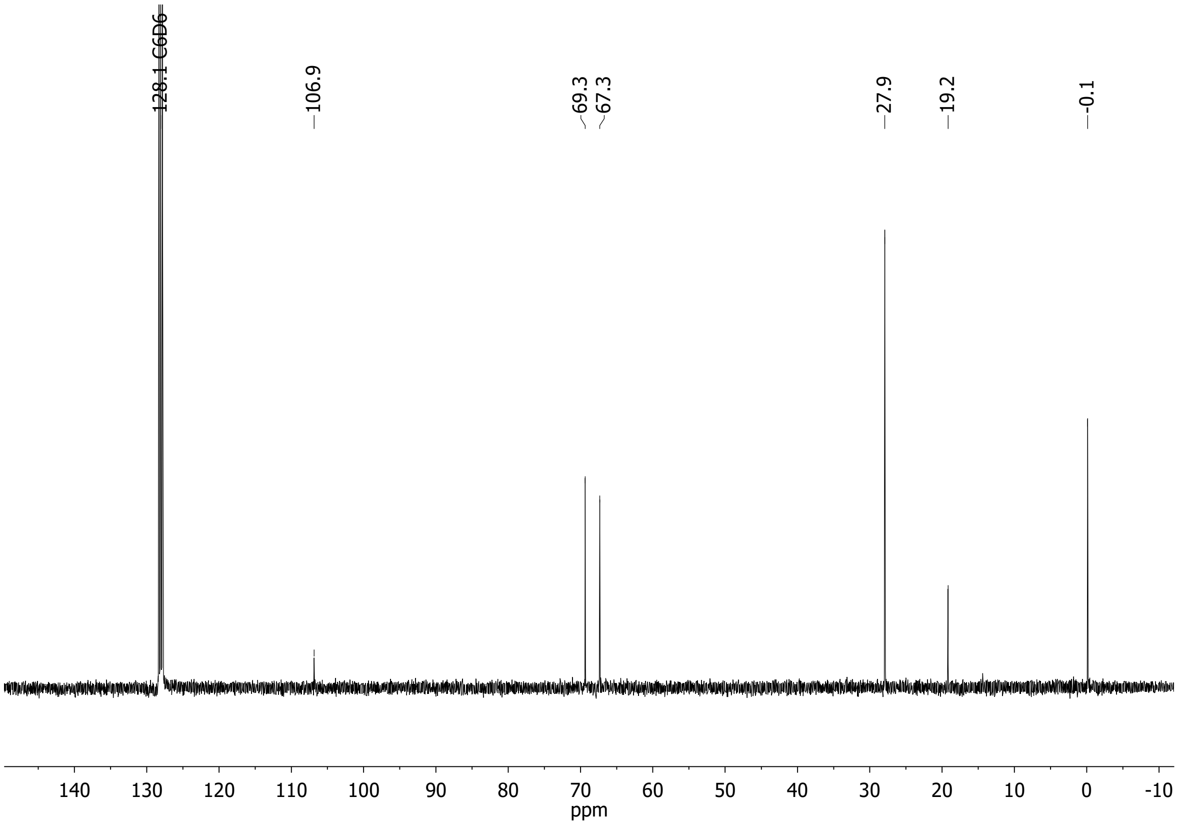

Figure S23. $^{13}C\{^{1}H\}$ NMR spectrum (101 MHz, C_6D_6) of **24**.

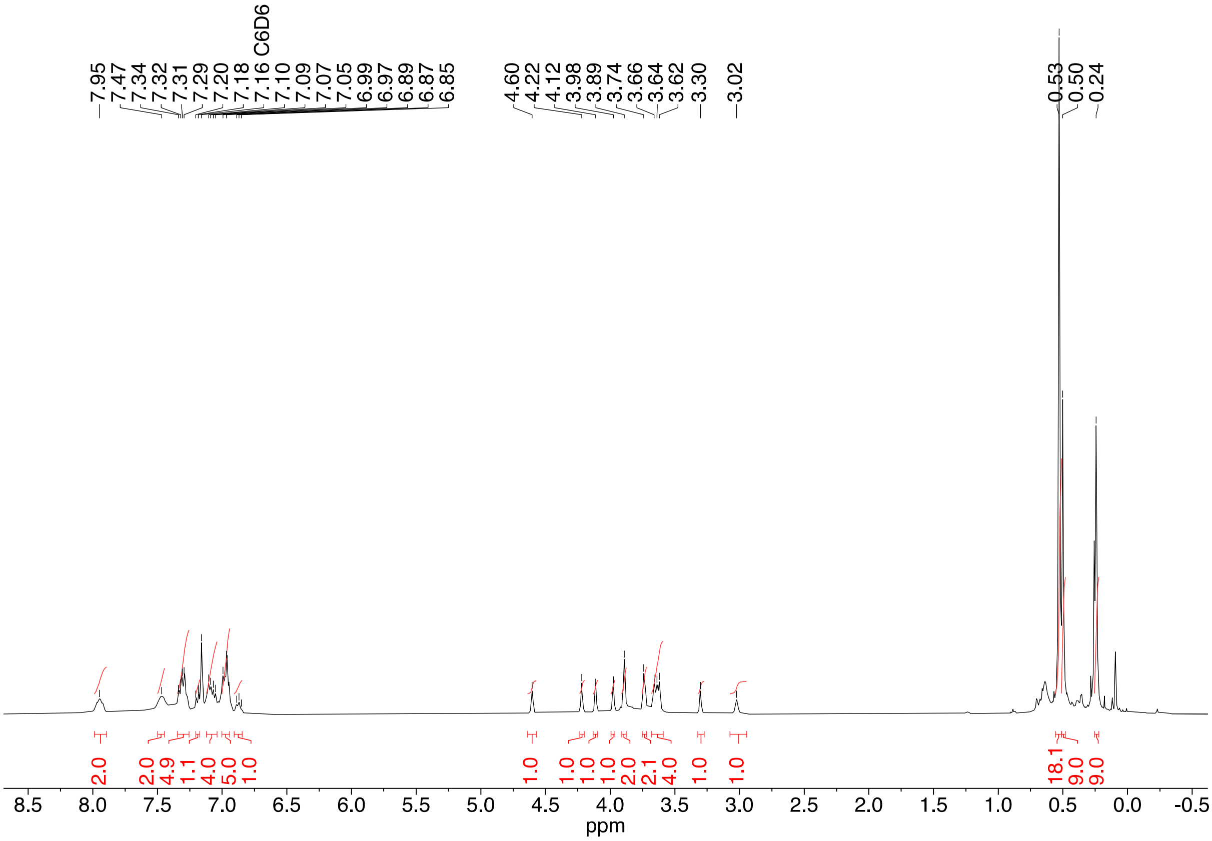

Figure S24. ^{1}H NMR spectrum (400 MHz, C_6D_6) of **25**.

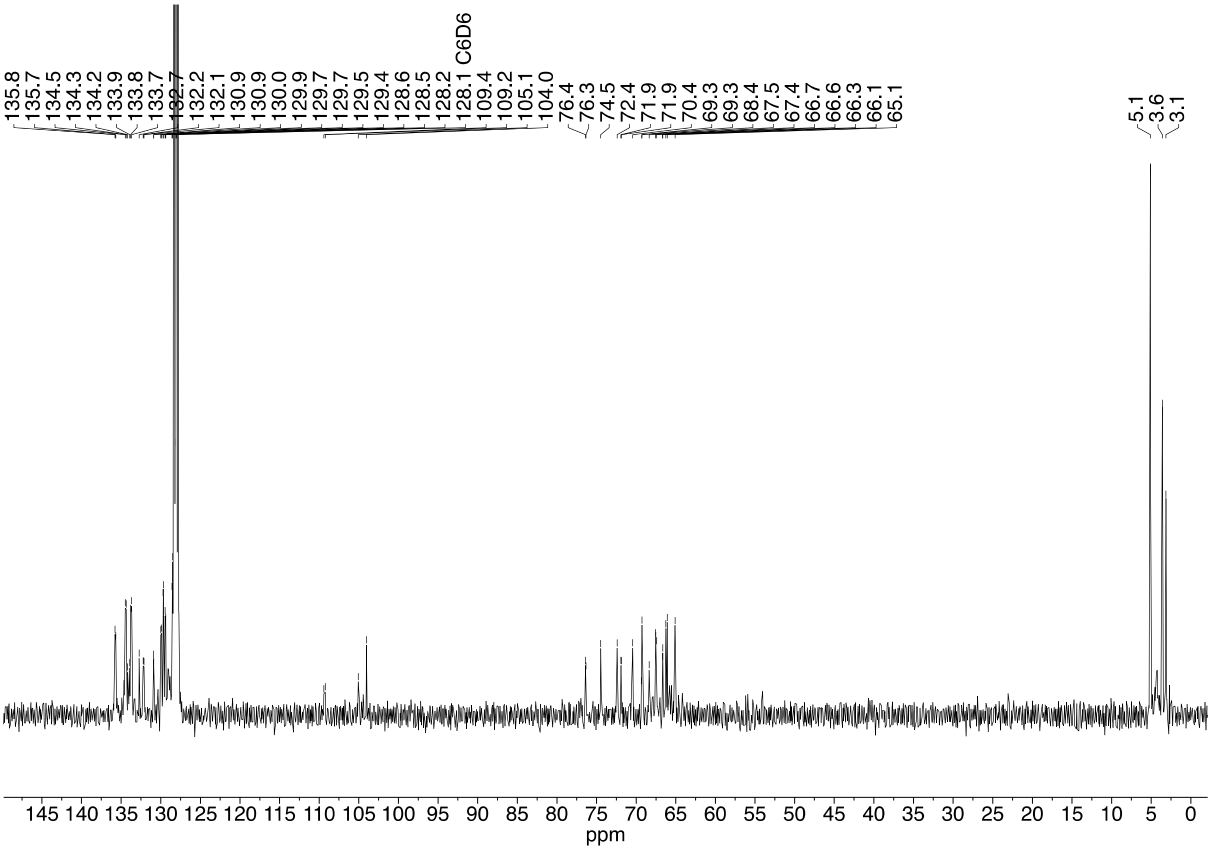

Figure S25. $^{13}C\{^1H\}$ NMR spectrum (101 MHz, C_6D_6) of **25**.

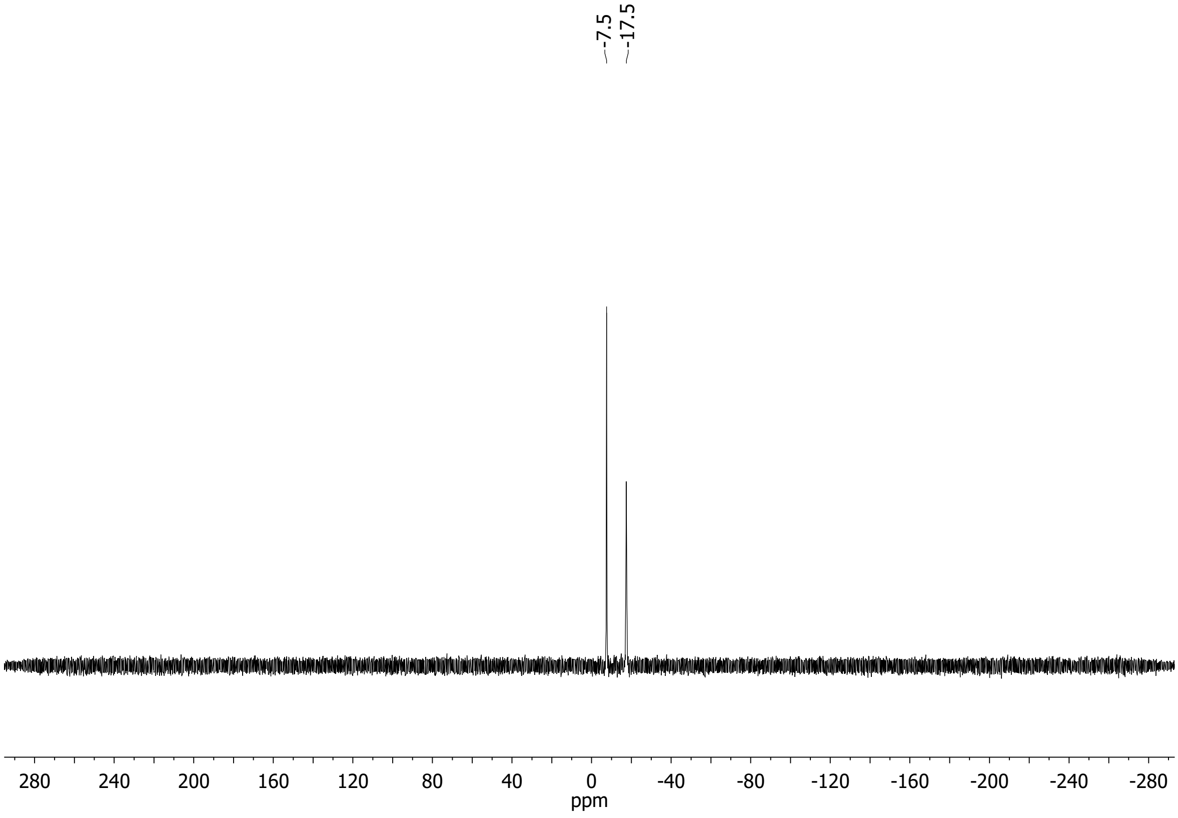

Figure S26. $^{31}P\{^1H\}$ NMR spectrum (202 MHz, C_6D_6) of **25**.

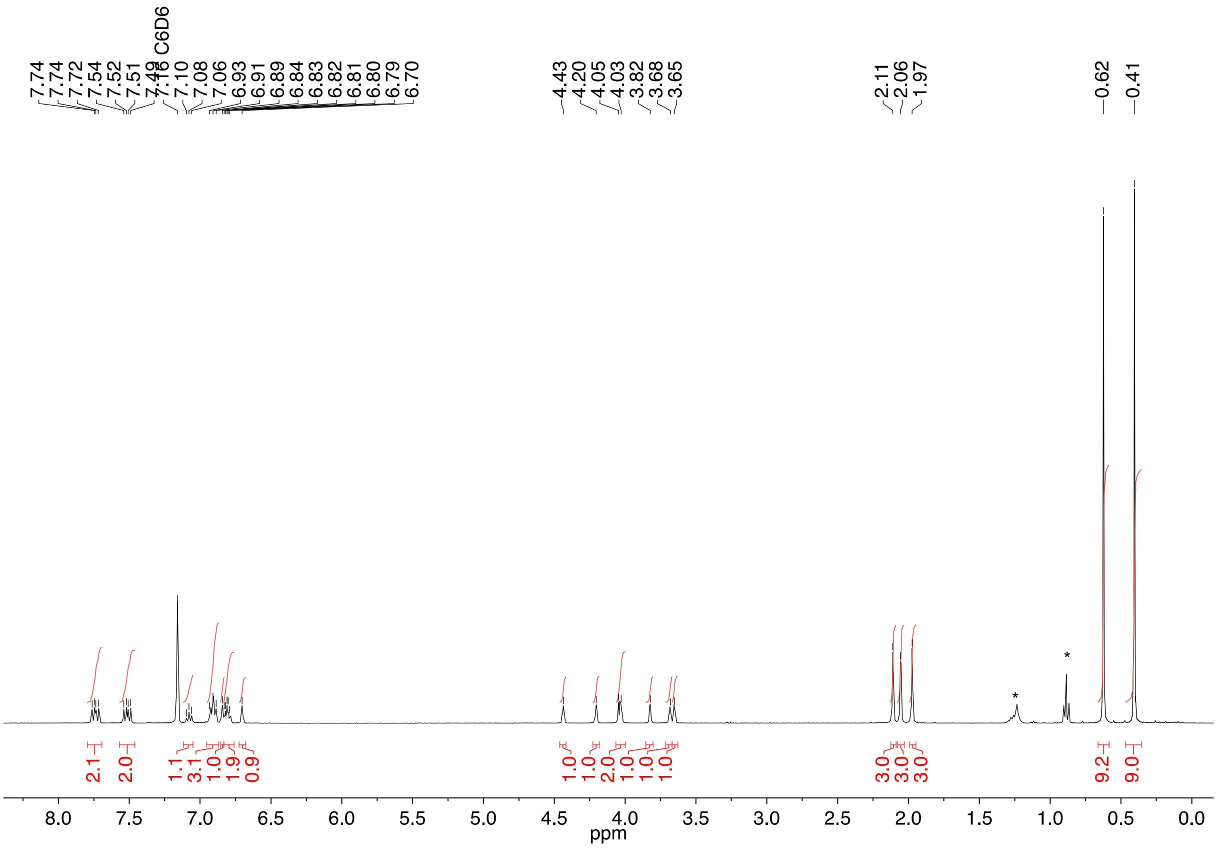

Figure S27. ^{1}H NMR spectrum (400 MHz, C_6D_6) of **26**. Signals marked (*) belong to residual *n*-hexane.

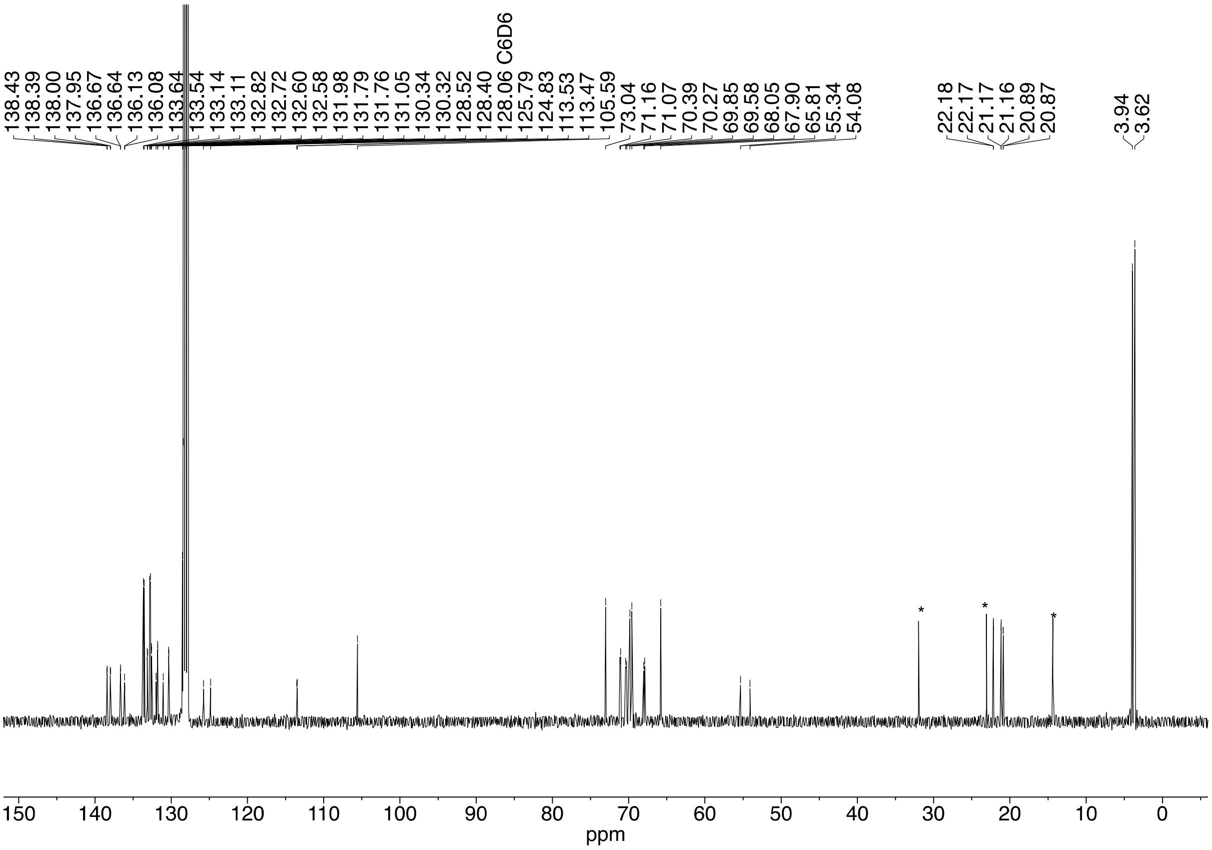

Figure S28. $^{13}C\{^{1}H\}$ NMR spectrum (101 MHz, C_6D_6) of **26**. Signals marked (*) belong to residual *n*-hexane.

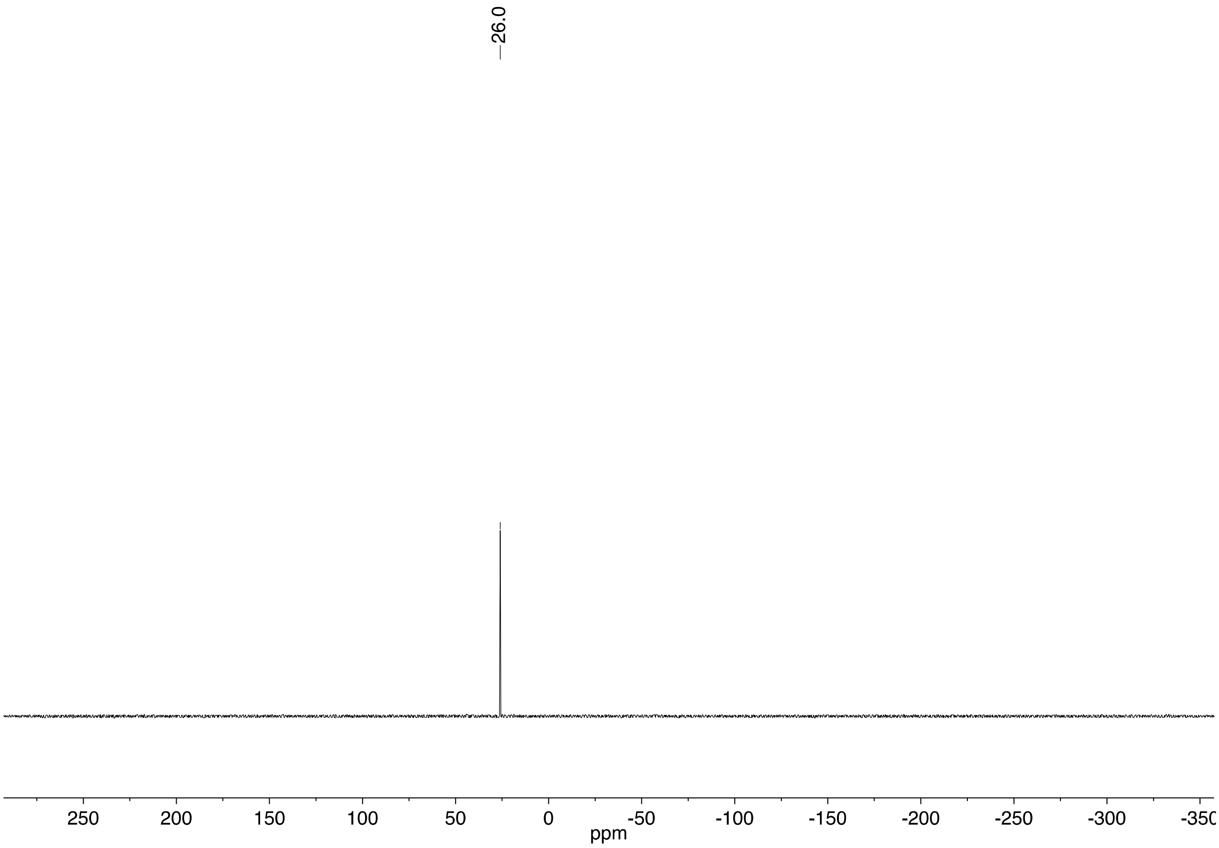

Figure S29. $^{31}P\{^{1}H\}$ NMR spectrum (202 MHz, C_6D_6) of **26**.
